# Unligated Okazaki Fragments Induce PCNA Ubiquitination and a Requirement for Rad59-Dependent Replication Fork Progression

**DOI:** 10.1371/journal.pone.0066379

**Published:** 2013-06-18

**Authors:** Hai Dang Nguyen, Jordan Becker, Yee Mon Thu, Michael Costanzo, Elizabeth N. Koch, Stephanie Smith, Kyungjae Myung, Chad L. Myers, Charles Boone, Anja-Katrin Bielinsky

**Affiliations:** 1 University of Minnesota, Department of Biochemistry, Molecular Biology and Biophysics, Minneapolis, Minnesota, United States of America; 2 Banting and Best Department of Medical Research, The Donnelly Centre, University of Toronto, Toronto, Ontario, Canada; 3 Department of Molecular Genetics, University of Toronto, Toronto, Ontario, Canada; 4 University of Minnesota, Department of Computer Science and Engineering, Minneapolis, Minnesota, United States of America; 5 Genome Instability Section, Genetics and Molecular Biology Branch, National Human Genome Research Institute, National Institutes of Health, Bethesda, Maryland, United States of America; Universita’ di Milano, Italy

## Abstract

Deficiency in DNA ligase I, encoded by *CDC9* in budding yeast, leads to the accumulation of unligated Okazaki fragments and triggers PCNA ubiquitination at a non-canonical lysine residue. This signal is crucial to activate the S phase checkpoint, which promotes cell cycle delay. We report here that a *pol30-K107* mutation alleviated cell cycle delay in *cdc9* mutants, consistent with the idea that the modification of PCNA at K107 affects the rate of DNA synthesis at replication forks. To determine whether PCNA ubiquitination occurred in response to nicks or was triggered by the lack of PCNA-DNA ligase interaction, we complemented *cdc9* cells with either wild-type DNA ligase I or a mutant form, which fails to interact with PCNA. Both enzymes reversed PCNA ubiquitination, arguing that the modification is likely an integral part of a novel nick-sensory mechanism and not due to non-specific secondary mutations that could have occurred spontaneously in *cdc9* mutants. To further understand how cells cope with the accumulation of nicks during DNA replication, we utilized *cdc9-1* in a genome-wide synthetic lethality screen, which identified *RAD59* as a strong negative interactor. In comparison to *cdc9* single mutants, *cdc9 rad59Δ* double mutants did not alter PCNA ubiquitination but enhanced phosphorylation of the mediator of the replication checkpoint, Mrc1. Since Mrc1 resides at the replication fork and is phosphorylated in response to fork stalling, these results indicate that Rad59 alleviates nick-induced replication fork slowdown. Thus, we propose that Rad59 promotes fork progression when Okazaki fragment processing is compromised and counteracts PCNA-K107 mediated cell cycle arrest.

## Introduction

Replication fork arrest in response to DNA lesions, such as UV-induced thymine dimers that physically block DNA synthesis and lead to exposure of unreplicated, single-stranded (ss) DNA has been studied extensively in multiple different model organisms [1]. However, how cells monitor the integrity of replication intermediates that undergo Okazaki fragment processing is less well understood. Given that human cells produce on the order of 30 million Okazaki fragments that need to be processed and ligated during a single round of replication, a tracking system should be in place to account for possible errors that could lead to the accumulation of nicked DNA. The importance of such a surveillance system is underscored by mutations impinging on proper Okazaki fragment processing that have been identified in human cancer patients and whose cancer-causing effect has been recapitulated in animal studies [2], [3]. In particular, a DNA ligase I-deficiency causes not only growth retardation similar to other replication-associated genetic syndromes but also lymphoma [3].

DNA ligase I catalyzes the sealing of nicks between adjacent 3′-OH and 5′-PO_4_ termini and is crucial for DNA replication, repair and recombination. The DNA ligation mechanism involves three nucleotidyl transfer reactions [4]. In the first step of the ligation reaction, DNA ligase reacts with either ATP or NAD^+^ (in prokaryotes) to form a ligase-adenylate intermediate where 5′-adenosine monophosphate (AMP) is linked by a phosphoamide bond with the lysine residue in the active site. In the second step, AMP is transferred to the 5′-PO_4_ terminus of the nick to form a DNA-adenylate. Finally, DNA ligase catalyzes the nucleophilic attack of the 3′-OH to the DNA-adenylate to covalently join the two ends of the DNA strands and release AMP.

The budding yeast *S. cerevisiae* encodes two different DNA ligases, Cdc9 and Dnl4, which are homologs of human DNA ligases I and IV, respectively [5]–[7]. Given their different substrate specificities, the two proteins have clearly distinct roles in DNA metabolism and cannot substitute for each other [6], [7]. Whereas Dnl4 functions in double strand break (DSB) repair via non-homologous end joining (NHEJ), Cdc9 participates in base excision repair (BER) and nucleotide excision repair (NER) [4]. Additionally, Cdc9 is essential for the ligation of Okazaki fragments and interacts genetically and physically with many proteins involved in Okazaki fragment maturation [4]. One such interacting protein is PCNA. The N-terminus of Cdc9 contains a conserved PCNA interacting peptide (PIP) box motif, QxxLxxFF, which facilitates its interaction with PCNA [8]. Deletion of the PIP-box in Cdc9 affects DNA repair, but not mitotic growth, suggesting that the interaction is not essential for Okazaki fragment maturation [9]. In agreement with these data, the smallest form of *Chlorella* virus DNA ligase, containing only the minimal core domain of DNA ligase, but not a PIP-box, complements *cdc9Δ* cells [9]. Unlike DNA ligase I, ChVLig has a very high intrinsic affinity for nicks and uses a “latch” that is absent in DNA ligase I. This latch enables ChVLig to encircle nicks without relying on the recruitment by other factors such as PCNA [10].

Temperature sensitive (ts) alleles of *CDC9* were isolated from the original screen for cell division cycle (cdc) mutants. At the restrictive temperature, *cdc9* mutants arrest in late S/G2 phase of the cell cycle and accumulate unligated Okazaki fragments that are joined upon shifting to permissive conditions [11]. This suggested that cells replicate the whole genome leaving nicks behind for repair in G2 prior to entry into mitosis [12]. The G2 arrest of *cdc9ts* mutants is controlled by Rad9-dependent phosphorylation of the S phase checkpoint kinase Rad53, which is triggered when nicks are converted to DSBs [13], [14]. However, we reported recently that the absence of DNA ligase I in more stringent *cdc9ts* alleles caused a delay in S phase progression and activation of the S phase checkpoint kinase Rad53, which was mediated through both Mrc1, the mediator of the replication checkpoint [15], and Rad9 [16]. This indicated that not only DSBs were present in *cdc9ts* mutants, but also stalled replication forks [16], [17]. Importantly, robust activation of Rad53 in *cdc9ts* mutants required PCNA ubiquitination at a novel residue, K107 rather than K164, the well-known conserved site, which is ubiquitinated in response to DNA damaging agents such as UV-irradiation or methyl methanesulfonate (MMS) that induce fork stalling [16], [18]. We hypothesized that cells can distinguish the DNA structures arising from nicked DNA due to DNA ligase I deficiency *versus* extended ssDNA regions caused by UV-irradiation or MMS exposure [2]. How cells cope with the accumulation of such putative nicked replication intermediates and promote S phase progression is unknown. Several studies demonstrated that the inhibition of DNA ligase I activity in both yeasts and humans results in a higher incidence of DNA recombination [3], [19], [20]. In budding yeast, this notion is supported by the synthetic lethality between a recombination deficient mutant, *rad52*, and *cdc9* [5]. These results are consistent with the assumption that DSBs are formed in *cdc9ts* mutants that require repair by homologous recombination (HR).

*RAD52* is essential to promote DSB repair by HR, which is divided into two sub-categories, *RAD51*-dependent and *RAD51*-independent pathways [21]. *RAD51*-dependent HR repairs most DSBs in mitotic cells by initiating strand invasion of a 3′-ssDNA tail following the formation of a Rad51 filament [22]. The alternative *RAD51*-independent pathway involves a single strand annealing (SSA) step, mediated by *RAD52* and *RAD59* for DSB repair between direct repeat sequences and in break-induced replication [21], [22].

In this study, we report that the accumulation of either “clean” (3′-OH and 5′-PO_4_ termini) or “dirty” (DNA-adenylate; 3′-OH and 5′-AMP ends) nicks in *cdc9-1* mutants triggered PCNA mono- and poly-ubiquitination at K107. Moreover, K107 ubiquitination was responsible for causing a delay in S phase progression. To identify pathways involved in nick resolution, we performed a synthetic genetic array (SGA) screen with *cdc9-1* mutants and verified results by selected manual tetrad dissections. Besides the known requirement for genes involved in DSB repair via *RAD51/RAD52*-mediated HR, we uncovered strong genetic interactions with components of the *RAD51*-independent SSA pathway, comprising *RAD59*, *RAD1*, and *RAD10*. Surprisingly however, deletion of *SLX4* (synthetically lethal with *sgs1*), a crucial component of SSA-mediated DSB repair [23], [24], did not affect *cdc9-1* mitotic growth. These results suggested that SSA might be dispensable for DSB repair in *cdc9-1* cells. This was further corroborated by the fact that the combined deletion of *RAD59* and *RAD1* had a more severe effect on the survival of *cdc9* mutants than deletion of *RAD59* alone, arguing that the two genes did not act in the same pathway. Targeted analysis of *RAD59* further revealed that its ablation in *cdc9* mutants resulted in enhanced Mrc1 phosphorylation. We concluded that stalled replication forks accumulated more frequently in *cdc9 rad59Δ* double than *cdc9* single mutants. Together, these results uncover a role for non-canonical PCNA ubiquitination in facilitating S phase delay and for *RAD59* in promoting slow fork progression when DNA ligase I is limiting.

## Results

### Accumulation of Nicked DNA due to DNA Ligase I Deficiency Triggers PCNA Ubiquitination Independently of Lysine 164

The depletion of Cdc9 in *S. cerevisiae* drastically slows down S phase progression [2]. Specifically, the loss of DNA ligase I triggers a novel ubiquitination pathway that targets PCNA at a lysine residue distinct from K164 and acts upstream of S phase checkpoint activation [16]. However, it was still unclear what molecular defect caused PCNA ubiquitination in *cdc9* mutants. We envisioned three possible scenarios that could arise when DNA ligase I is limiting. First, cells could directly sense the accumulation of nicks; second, cells might recognize the absence of the PCNA-Cdc9 interaction or third, because *cdc9* mutants are known to be highly mutagenic [5], secondary defects unrelated to the generation of nicks could cause the ubiquitination of PCNA.

**Figure 1:**
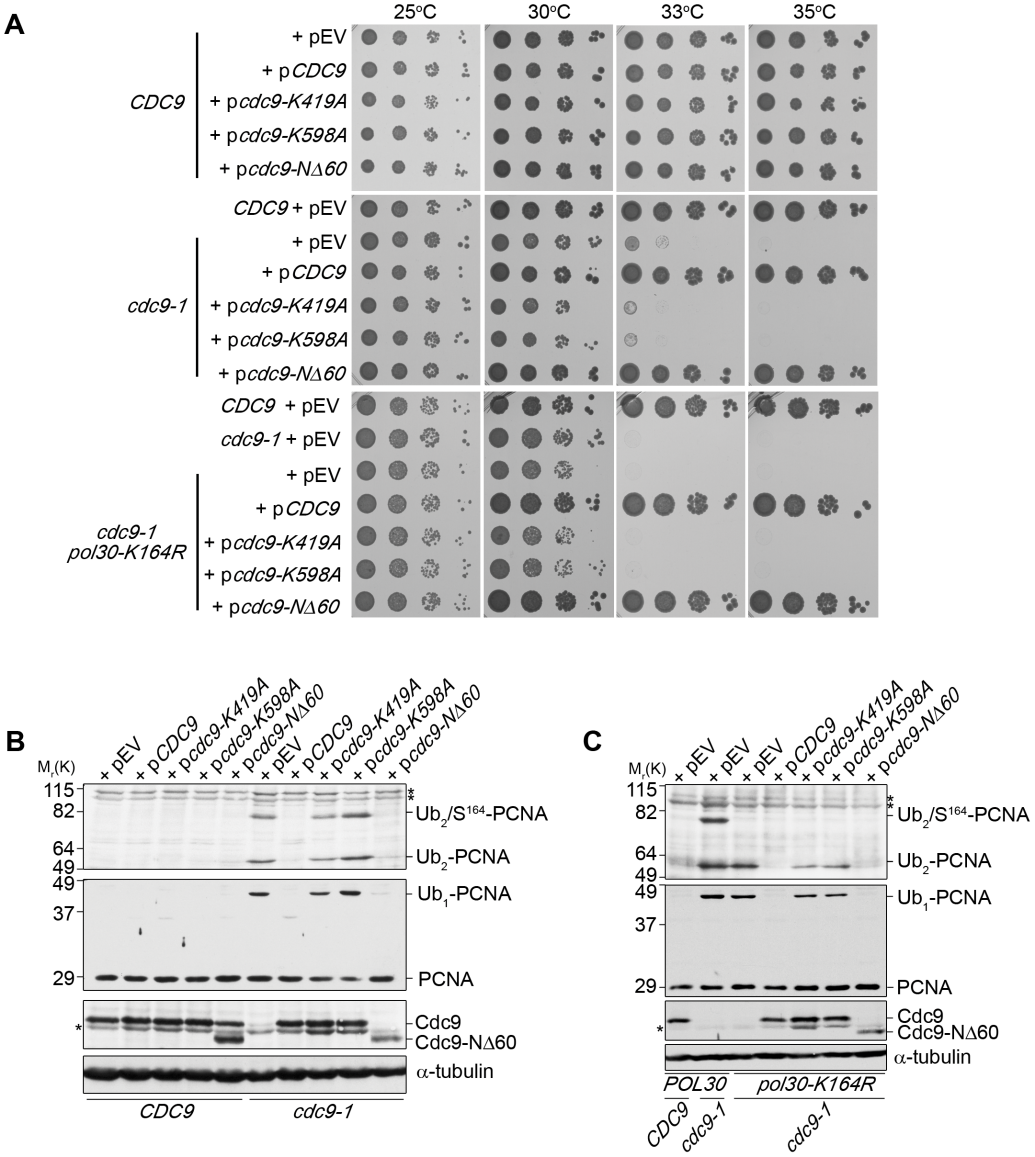
Defects in DNA ligase I trigger PCNA mono- and poly-ubiquitination independently of lysine 164. (**A**) Successive 10-fold dilutions of the indicated strains were grown on Sc-His plates for 3 days at the indicated temperatures. (**B, C**) All strains shown in **A** were grown asynchronously to mid-log phase at 25°C and shifted to the restrictive temperature of 35°C for 3 hr. PCNA and its ubiquitinated forms, and Cdc9 were detected with anti-PCNA (S871) and anti-Cdc9 antibodies, respectively. α-tubulin served as a loading control. Asterisks indicate non-specific bands.

To distinguish between these different scenarios, we complemented *cdc9-1*, and *cdc9-1 pol30-K164R* (PCNA-K164R) cells with plasmids expressing either wild-type or mutant Cdc9 under its endogenous promoter. As a control, these plasmids were also introduced into wild-type cells (Figure 1). As expected, expression of wild-type *CDC9* rescued the temperature sensitivity of *cdc9-1* mutants at 35°C (Figure 1A). In addition, we introduced two plasmids carrying DNA ligase I mutations in the active center of the enzyme, p*cdc9-K419A* and p*cdc9-K598A*. K419 is critical for the covalent binding of AMP in the first step of ligation and mutation of this residue results in the accumulation of “clean” nicks, whereas K598 is important for the final DNA de-adenylation step, and substitution of this residue with alanine results in the accumulation of “dirty” nicks [9], [25], [26]. Neither of the two catalytically inactive Cdc9 mutants allowed *cdc9-1* cells to grow at the restrictive temperature (Figure 1A). In contrast, a truncated version of Cdc9 lacking the PIP-box motif (p*cdc9-NΔ60*), which is required for the interaction with PCNA [26], fully rescued *cdc9-1* cells at 35°C. Lastly, *cdc9-1* mutants expressing PCNA-K164R (*cdc9-1 pol30-K164R*), which renders cells sensitive to UV-irradiation and MMS [18], did not exhibit any additional temperature sensitivity as compared to *cdc9-1* single mutants (Figure 1A). These results indicated that the survival of *cdc9-1* mutants did not depend on K164 of PCNA, but rather on the reconstitution of DNA ligase I activity.

In parallel to testing cell viability, we also monitored DNA ligase I expression levels and the status of PCNA modification at 35°C (Figures 1B and 1C). The PCNA banding pattern is rather complex, but our previous work showed that PCNA is ubiquitinated at K107, and sumoylated at either K127 or K164 in *cdc9-1* mutants [2], [16]. Consistent with these earlier data [16], we detected four different PCNA species in *cdc9-1* cells that carried an empty vector (Figure 1B): unmodified PCNA (29 kDa), mono-ubiquitinated PCNA (39 kDa), and putatively poly-ubiquitinated PCNA (at ∼52 and 76 kDa). However, it was possible that a single PCNA monomer could be simultaneously sumoylated (at K164) and ubiquitinated (at K107). Indeed, a K164R substitution in PCNA diminished the 76 kDa band in *cdc9-1* cells (Figure 1C, compare lanes 2 and 3). Thus, the 76 kDa band most likely represented PCNA that was di-ubiquitinated at K107 and sumoylated at K164 (marked as Ub_2_/S^164^-PCNA in Figures 1B and 1C). In contrast, the 39 kDa and ∼52 kDa bands represented solely mono- and di-ubiquitinated PCNA species, respectively (marked as Ub_1_-PCNA and Ub_2_-PCNA in Figures 1B and C), as reported [16].

**Figure 2:**
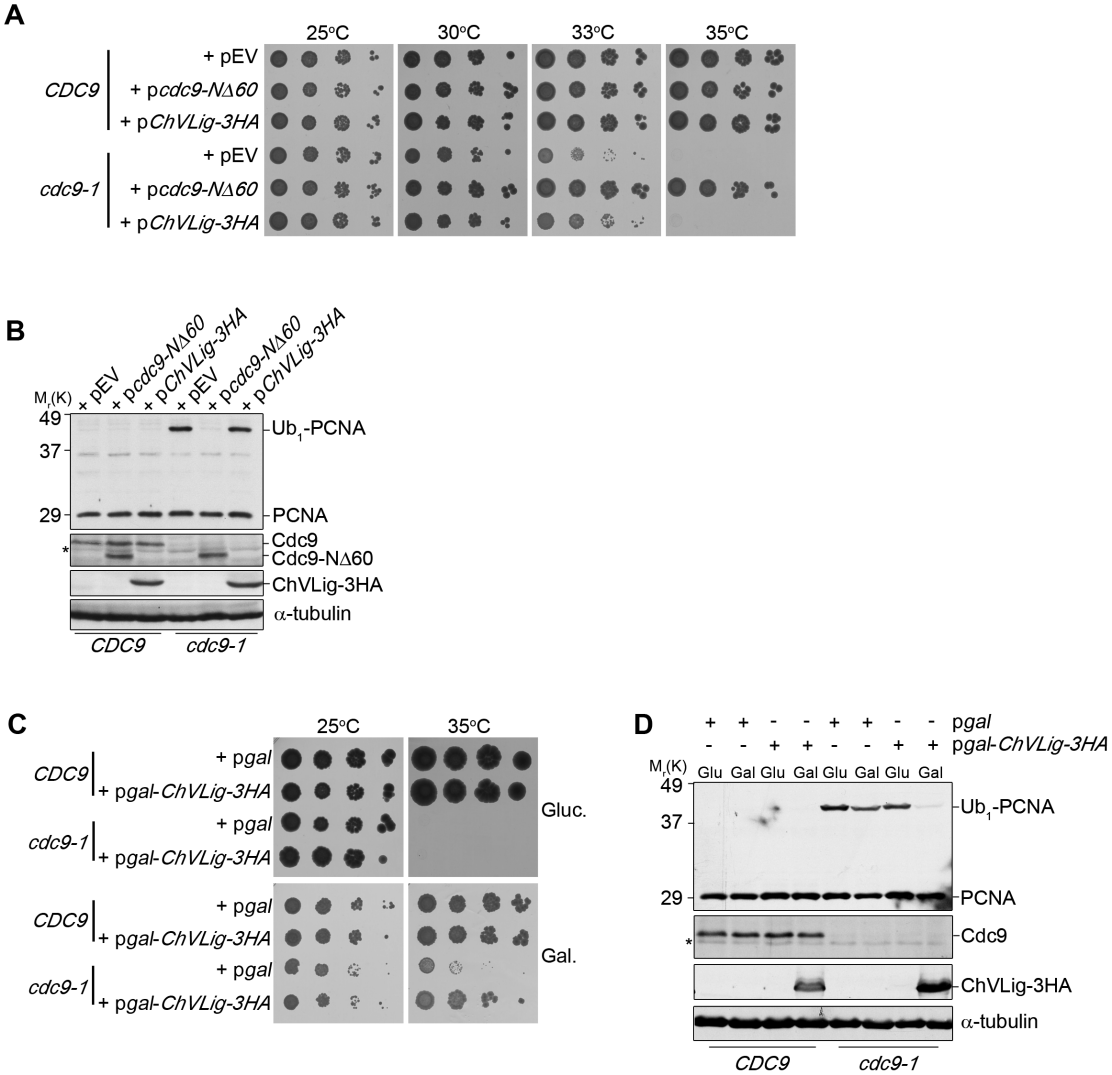
Overexpression of *Chlorella* virus DNA ligase fully complements *cdc9-1* temperature sensitivity and reverses PCNA ubiquitination. (**A**) Successive 10-fold dilutions of the indicated strains were grown on Sc-His plates for 3 days at the indicated temperatures. Expression of *Chlorella* virus DNA ligase from the p*ChVLig-3HA* plasmid was under the control of the *CDC9* promoter. (**B**) All strains shown in **A** were grown asynchronously to mid-log phase at 25°C. Subsequently, cultures were shifted to the restrictive temperature of 35°C for 3 hr. PCNA and its ubiquitinated forms, Cdc9 and ChVLig-3HA were detected with anti-PCNA (S871), anti-Cdc9, and anti-HA antibodies, respectively. (**C**) Successive 10-fold dilutions of the indicated strains were grown on Sc-His plates containing either 2% glucose or 2% galactose for 5 days at 25°C and 35°C. Overexpression of *Chlorella* virus DNA ligase from the pRS423gal-*ChVLig-3HA* plasmid was under the control of the *GAL10* promoter. (**D**) Yeast strains shown in **C** were grown asynchronously to mid-log phase in medium containing 2% raffinose at 25°C. Cultures were split and grown in the presence of either 2% glucose or 2% galactose at the restrictive temperature of 35°C for 3 hr. PCNA and its ubiquitinated forms, Cdc9 and ChVLig-3HA were detected with anti-PCNA (S871), anti-Cdc9, and anti-HA antibodies, respectively. In **B** and **D**, α-tubulin served as a loading control. Asterisks indicate non-specific bands.

When we complemented *cdc9-1* cells with wild-type Cdc9 (*cdc9-1*+ p*CDC9*), both PCNA mono- and poly-ubiquitination were abolished (Figure 1B). Because PCNA ubiquitination is readily detectable at 25°C [16], the disappearance of the ubiquitinated PCNA molecules presented a true reversal of the ubiquitination response. This result allowed us to exclude nonspecific, secondary effects as a trigger of PCNA ubiquitination. Moreover, expression of the Cdc9 PIP-box mutant (*cdc9-1*+ p*cdc9-NΔ60*) also reversed PCNA mono- and poly-ubiquitination, making it highly unlikely that cells recognized the absence of PCNA-Cdc9 interaction (Figure 1B). To further corroborate this notion, we examined the ability of *Chlorella* virus DNA ligase (ChVLig) to complement the DNA ligase I deficiency in yeast. ChVLig is the smallest known ATP-dependent ligase [27], containing only a conserved catalytic core that consists of a nucleotidyltransferase (NTase) and an oligonucleotide/oligosaccharide binding (OB)-fold domain [10]. Since ChVLig has no additional domains beyond its catalytic NTase-OB core, it was previously suggested that the protein can not interact with the eukaryotic replication machinery [9]. When we expressed *ChVLig*-*3HA* under the control of the *CDC9* promoter, it only partially rescued the temperature sensitivity of *cdc9-1* mutants (*cdc9-1*+ p*ChVLig-3HA*) at 33°C, but not at 35°C at which temperature PCNA mono-ubiquitination remained visible (Figures 2A and B). However, upon overexpression of ChVLig-3HA from a galactose-inducible promoter we rescued *cdc9-1* temperature sensitivity and reversed PCNA mono-ubiquitination (Figures 2C and D). Thus, the ligation of nicks appeared to eliminate PCNA ubiquitination in the absence of a PCNA-DNA ligase interaction. This was further substantiated by the observation that PCNA ubiquitination remained unchanged in *cdc9-1* cells that were complemented with either p*cdc9-K419A* or p*cdc9-K598A*, encoding two different catalytically inactive forms of DNA ligase I, which retain PCNA binding activity (Figure 1B). Importantly, similar alterations to the ubiquitination pattern of PCNA (Ub_1_-PCNA and Ub_2_-PCNA) were observed in *cdc9-1 pol30-K164R* mutants after complementation with different DNA ligase I constructs (Figure 1C). These data unequivocally demonstrate that cells induce PCNA mono- and poly-ubiquitination independently of K164 in response to nicked DNA. In summary, based on our previous study [16] and these new results, we postulate that PCNA ubiquitinated at K107 (PCNA^K107-Ub^) functions as a nick sensor at replication forks in budding yeast.

### PCNA Ubiquitination at K107 is Important for the S Phase Delay in *cdc9-1* Cells

**Figure 3:**
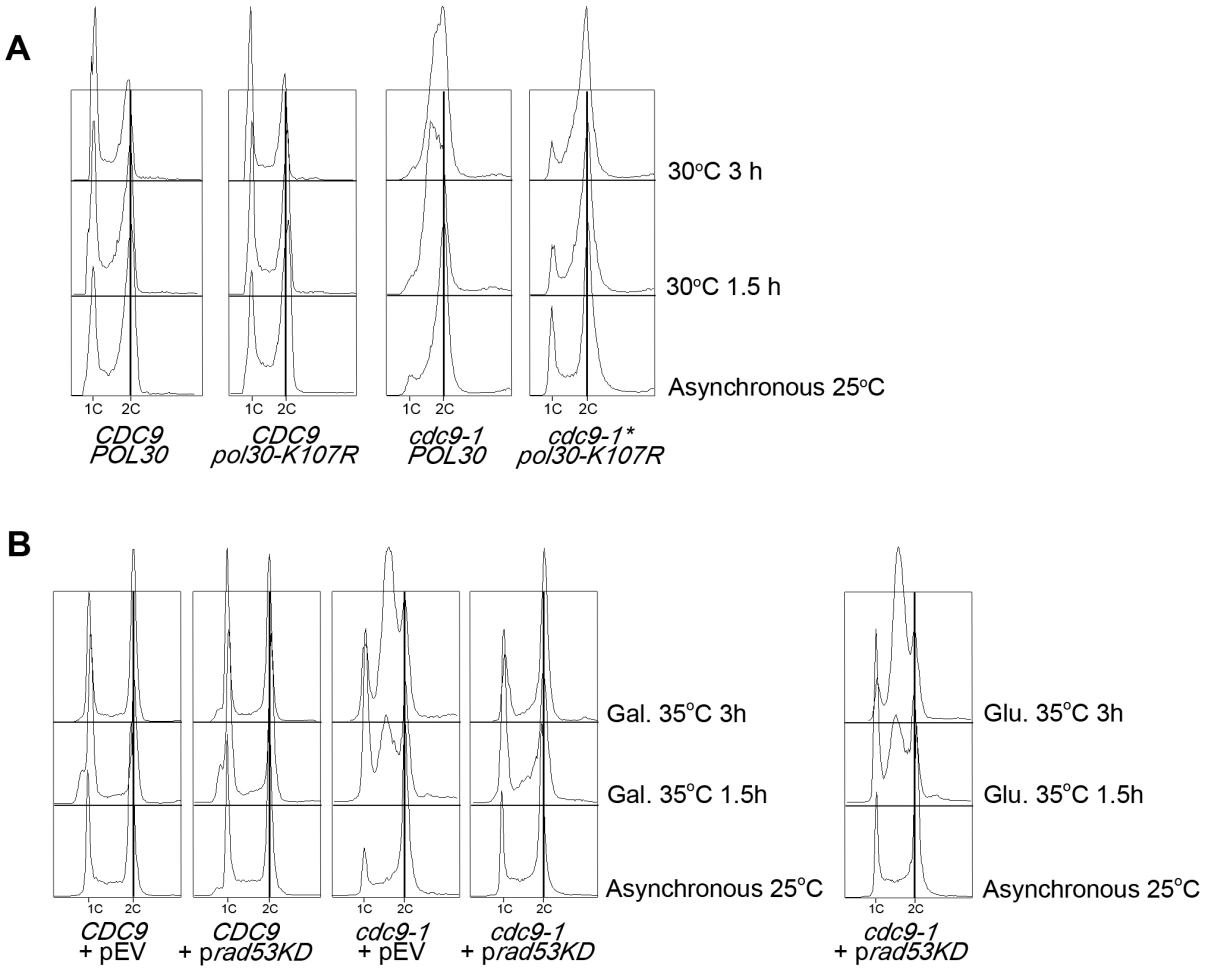
PCNA ubiquitination at K107 is crucial for the S phase arrest in *cdc9-1* mutants. (**A**) Asynchronous cultures of the indicated strains were grown at 25°C and shifted to 30°C for 1.5 and 3 hr. (**B**) Asynchronous cultures of the indicated strains were grown at 25°C and shifted to 35°C for 1.5 and 3 hr in the presence of 2% galactose. On the right, the same asynchronous *cdc9-1*+ p*Rad53-KD* culture was split and grown in the presence of 2% glucose. In **A** and **B**, DNA content was stained with Sytox Green and monitored by flow cytometry. The vertical line indicates a 2C DNA content.

PCNA^K107-Ub^ is a prerequisite to activate the S phase checkpoint kinase Rad53 in *cdc9ts* mutants [16]. We previously reported that most *cdc9-1 pol30-K107R* double mutants were synthetically lethal after tetrad dissection. Nevertheless, we isolated a viable double mutant that showed slightly increased DNA ligase I levels, designated as cdc9-1* pol30-K107R [16]. Temperature sensitivity of this mutant can be rescued by the overexpression of *cdc9-1* (Figure S1), arguing that the primary defect is still linked to the DNA ligase I deficiency. We predicted that cdc9-1* pol30-K107R mutants would readily progress through S phase, since robust Rad53 activation was drastically reduced in *pol30-K107R* mutants upon DNA ligase I depletion [16]. Therefore, we monitored cell cycle progression of *cdc9-1*, cdc9-1* pol30-K107R, and their respective parental strains (Figure 3A). Because cdc9-1* pol30-K107R mutants are much more temperature sensitive than *cdc9-1* cells (Figure S1 and [16]), we performed the temperature shift experiments at 30°C instead of 35°C. Neither *CDC9* nor *CDC9 pol30-K107R* strains exhibited any cell cycle defects at 30°C (Figure 3A). However, *cdc9-1* cells accumulated in, and progressed through, S phase very slowly after 1.5 and 3 hr temperature shifts, resulting in a broad S phase peak. In contrast, cdc9-1* pol30-K107R mutants did not exhibit a broad S phase distribution and accumulated mostly in G2 (Figure 3A). These results support the notion that PCNA^K107-Ub^ facilitates robust activation of Rad53 [16], which then promotes S phase delay. In line with this claim, galactose-induced overexpression of a dominant negative Rad53 kinase-dead mutant (Rad53-K221A/D339A) [28], [29] also completely suppressed the accumulation of *cdc9-1* cells in S phase (compare galactose on the left vs. glucose on the right in Figure 3B).

Because K107 of PCNA appeared to have an important role in S phase checkpoint activation when Cdc9 is limiting, we asked whether a K107R substitution exhibited any effect on the growth or DNA damage signaling in DNA ligase I-proficient cells. We tested UV- and γ-irradiation as well as MMS, but did not detect any growth sensitivity (Figure S2A). In parallel, we also determined the rate of spontaneous mutations by testing resistance to canavanine. In this assay, the K107R mutant displayed a slightly elevated mutation frequency comparable to that of K164R and K183R mutants (Figure S2B). These results are consistent with the idea that PCNA ubiquitination at K107 is specific to nicked replication intermediates that persist during lagging strand synthesis.

### Identification of the DNA Repair Network in *cdc9-1* Mutants

To identify other factors that either enhance or counteract the role of ubiquitinated PCNA at stalled replication forks, we conducted a SGA screen using the *cdc9-1* mutant as the query strain, which was mated with approximately 4000 array strains carrying single deletions of non-essential genes [30]. The fitness of the double mutants was scored quantitatively by colony size at the semi-permissive temperature of 30°C [31]. We observed synthetic sickness between *cdc9-1* and *rad9Δ* (Table S3), in accordance with the documented role of *RAD9* in response to DNA damage [12], [13]. Since Rad9 associates with DSBs [14], the synthetic sickness of the *cdc9-1 rad9Δ* double mutants could indicate that some of the nicks in *cdc9-1* cells are converted to DSBs. Indeed, the rate for gross chromosomal rearrangements (GCRs) in *cdc9-1* mutants was 30-fold elevated over wild-type (Figure S3), indicative of the presence of spontaneous DSBs [32], [33].

The MRX (Mre11/Rad50/Xrs2) complex is the primary sensor of DSBs and crucial to initiate HR [21]. Consistent with the presence of DSBs in *cdc9-1* cells, we also identified synthetic sickness between *cdc9-1* and *mre11Δ* in our screen (Table S3). This is in line with the reported negative genetic interaction between *cdc9* and *rad50Δ* [34]. Curiously, *RAD52*, a major HR component showed no interaction with the *cdc9-1* allele in the SGA screen, although *cdc9 rad52* double mutants have been reported to be synthetically lethal [5]. Because *cdc9-1* mutants are highly mutagenic [5], we postulate that the *cdc9-1 rad52Δ* double mutants on the array likely accumulated a second site suppressor that allowed these mutants to grow at a similar rate as *cdc9-1* cells. To ensure that our *cdc9-1* strain exhibited the same genetic properties as described in the literature, we manually crossed it with *rad51Δ* and *rad52Δ* cells, respectively. As expected, we were not able to isolate any viable *cdc9-1 rad51Δ* or *cdc9-1 rad52Δ* double mutants, indicating that *RAD51*/*RAD52*-mediated HR is essential for survival (Figures S4A and B). Because our primary interest was the identification of novel factors that facilitate nick resolution and promote replication fork progression in *cdc9-1* cells, we focused on genes participating in pathways different from HR.

### Rad59 Plays a Crucial Role in *cdc9-1* Survival

**Figure 4:**
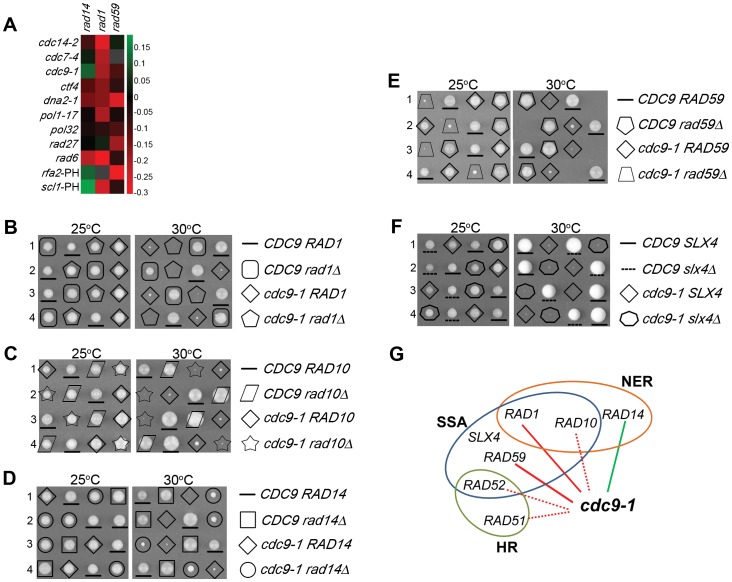
Validation of genetic interactions of *RAD1*, *RAD10*, *RAD14*, and *RAD59* with *cdc9-1* by tetrad dissection. (**A**) Heat map indicating strong positive (green) or negative (red) interactions between *cdc9-1* and *rad14Δ*, *rad1Δ* or *rad59Δ*, respectively. Black indicates no genetic interaction. Gray indicates that the interaction could not be scored. Among a collection of approximately 1800 SGA queries, only *scl1Δ* mutants displayed a genetic interaction signature similar to that of *cdc9-1* with respect to these three mutants. Other query mutants that exhibited similar genetic interactions with two out of the three genes are shown as well as *rad27Δ*, which exhibited synthetic sickness with *rad59Δ*. PH indicates strains received from Phil Hieter. The heat map was generated based on previously published [56] and new SGA screens in Table S2. (**B–F**) Selected diploid strains were dissected and incubated at either 25°C or 30°C. All haploid genotypes are as indicated on the right. Four independent tetrads (1-4) are laid out horizontally. The diploid strain genotypes are as followed: (**B**) *CDC9/cdc9-1 rad1Δ/RAD1*, (**C**) *CDC9/cdc9-1 rad14Δ/RAD14*, (**D**) *CDC9/cdc9-1 rad10Δ/RAD10*, (**E**) *CDC9/cdc9-1 rad59Δ/RAD59,* (**F**) *CDC9/cdc9-1 slx4Δ/SLX4*. (**G**) A Venn diagram summarizes some of the pertinent genetic interactions with *cdc9-1* mutants identified in this study. Genes are grouped into their respective repair pathways, homologous recombination (HR), HR-mediated single-strand annealing (SSA), and nucleotide excision repair (NER). Negative and positive genetic interactions with *cdc9-1* mutants identified in our SGA screen are illustrated as red and green solid lines, respectively. Red dotted lines indicate negative interactions with *cdc9-1* mutants that were only observed from manual tetrad analysis. No genetic interaction was observed between *SLX4* and *cdc9-1*.

**Figure 5:**
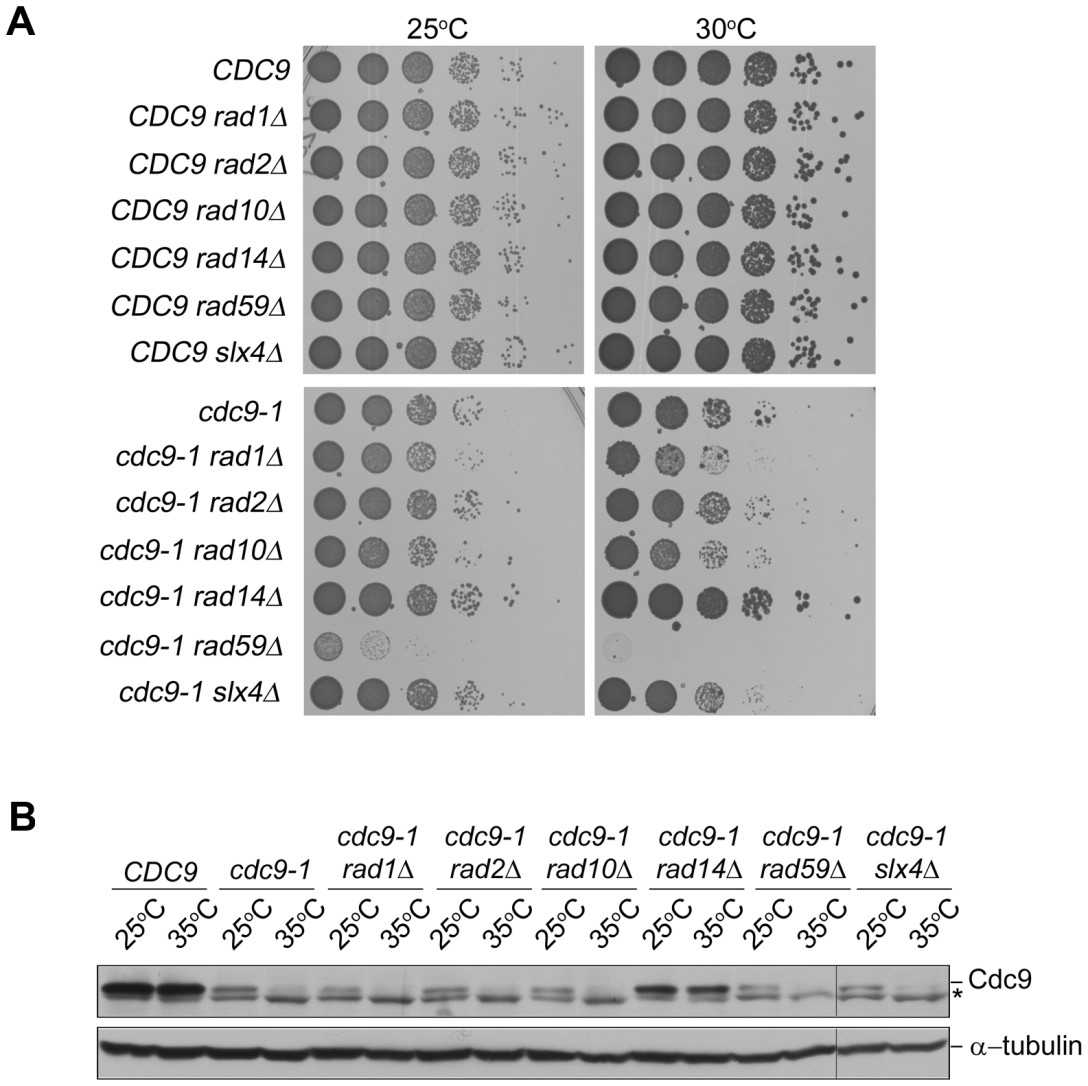
Spotting assay of different *cdc9-1* single and double mutants. (**A**) Successive 10-fold dilutions of the indicated strains were grown on YPD plates for 2 days at 25°C and 30°C. (**B**) Asynchronous cultures of the indicated strains were grown to mid-log phase at 25°C and subsequently shifted to the restrictive temperature of 35°C for 3 hr. Cdc9 expression was detected with anti-Cdc9 antibody and α-tubulin was used as a loading control. The asterisk indicates non-specific bands. Extracts from *cdc9-1 slx4Δ* mutants were fractionated on a separate gel, as indicated by the vertical lines.

**Figure 6:**
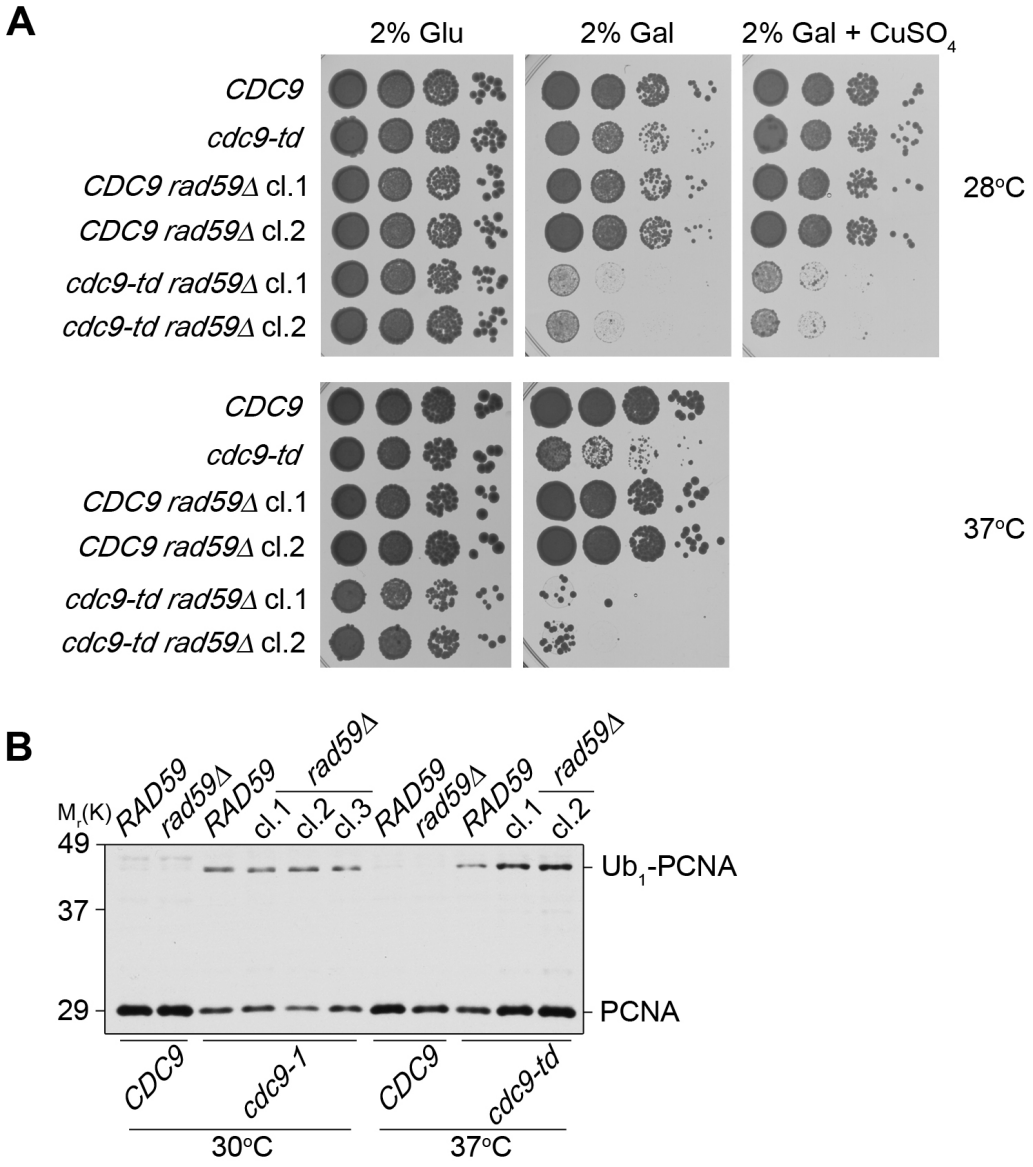
Deletion of *RAD59* in *cdc9* mutants does not affect PCNA mono-ubiquitination. (**A**) Successive 10-fold dilutions of the indicated strains were grown on YP plates containing either 2% glucose or 2% galactose at 28°C and 37°C. Cells were also spotted on YP +2% gal plates containing extra copper at 28°C to maintain wild-type Cdc9 expression in *cdc9-td* strains. Galactose induces the expression of *UBR1*, which promotes degradation of the heat-inducible degron fusion protein, Cdc9-td. (**B**) *cdc9 rad59Δ* double mutants were grown at the permissive temperature and subsequently shifted to the indicated temperatures for 3 hr. PCNA and its mono-ubiquitinated form were detected with anti-PCNA antibody (S871).

Besides genes involved in DSB repair, we observed that *cdc9-1* has genetic interactions with genes involved in NER, *RAD1* and *RAD14*. Strikingly, deletions of *RAD1* and *RAD14* showed drastically opposite effects (Figure 4A). Whereas the loss of *RAD1* decreased viability, the deletion of *RAD14* promoted cell growth. Rad1 interacts with Rad10 to form a structure-specific endonuclease, a homolog of the XPF/ERCC1 (for Xeroderma pigmentosum group F/Excision repair cross-complementing rodent repair deficiency, complementation group 1) complex in humans [35]. During NER, Rad14 recruits the Rad1-Rad10 endonuclease to the site of DNA damage to incise 5′ of the lesion [36]. Besides NER, the Rad1-Rad10 endonuclease has been implicated in the SSA pathway, a form of Rad51-independent repair that acts at DSBs and stalled replication forks between small repeat regions and is dependent on Rad59 [37], [38]. Indeed, we also observed a negative genetic interaction between *RAD59* and *cdc9-1* (Table S3). Based on these results, we hypothesized that *RAD1* and *RAD59* could possibly function in the same pathway in *cdc9-1* mutants and independently of *RAD14*. To validate the genetic interactions identified in the SGA screen, we constructed *cdc9-1 rad1Δ, cdc9-1 rad14Δ* and *cdc9-1 rad59Δ* double mutants (Figures 4B, D and E). Since *RAD1* interacts genetically and physically with *RAD10* in both NER and the SSA pathway (Figure 4G), we also generated *cdc9-1 rad10Δ* double mutants to perform tetrad dissections (Figure 4C). In parallel, we analyzed the growth behavior of the corresponding single and double mutants at 25°C and 30°C (Figure 5A). In addition to *RAD1*, *RAD10*, *RAD14* and *RAD59*, we deleted *RAD2*, encoding a ssDNA endonuclease required for NER [39], to assess whether it had a similar effect as *RAD14*. Since *cdc9-1 rad2Δ* cells mimicked *cdc9-1* single mutants, we excluded a role for NER. The subsequent analysis of DNA ligase I levels in all double mutants further revealed that *cdc9-1 rad14Δ* cells must have acquired a mutation that stabilized the enzyme and rendered it heat-resistant (Figure 5B). This easily explained the growth phenotype and was observed in two independent strains (data not shown). Consistent with the tetrad dissections, *cdc9-1 rad1Δ* and *cdc9-1 rad10Δ* strains displayed compromised growth at 30°C (Figure 5A), whereas we did not observe any viable *cdc9-1 rad59Δ* mutants at this temperature, suggesting that *RAD59* was necessary for the survival of *cdc9-1* cells at semi-permissive conditions (Figure 5A). Since *RAD1, RAD10* and *RAD59* have been indicated to function in SSA [21] (Figure 4G), we examined the genetic interaction between *cdc9-1* and *SLX4*, which is essential for DSB repair by SSA between direct repeats [23]. In SSA, Slx4 works independently of its binding partner Slx1 with which it forms a structure-specific endonuclease [23]. Three independent lines of evidence support the notion that Slx4 does not play any role in *cdc9* survival. First, we did not observe a genetic interaction between *cdc9-1* and *slx4Δ* by SGA. Second, in our manual tetrad dissection, we did not notice any differences in the colony size between *cdc9-1* single and *cdc9-1 slx4Δ* double mutants (Figure 4F). Lastly, *SLX4* deletion did not cause any growth inhibition of *cdc9-1* cells under either permissive or semi-permissive conditions (Figure 5A). Since SSA appeared to be dispensable in *cdc9* mutants, we predicted that Rad1/Rad10 did not function in this pathway either. To experimentally test this hypothesis, we attempted to compare *cdc9-1 rad59Δ* double mutants to *cdc9-1 rad1Δ rad59Δ* triple mutants. We were unable to generate the triple mutant in the *cdc9-1* background, and thus chose to perform the experiment with a *cdc9-td* strain. *cdc9-td* mutants express DNA ligase I when grown in glucose in the presence of copper, but they degrade the enzyme rapidly in the presence of galactose at elevated temperatures (Figures 6A and S5). If Rad1/Rad10 cooperated with Rad59, the double and triple mutants should have grown at very similar rates. However, we observed a 5-fold reduction in growth when *RAD1* was ablated from *cdc9-td rad59Δ* cells (Figure S5, see 5-fold dilutions at 37°C on 2% glucose plates). Taken together, we uncovered a strong requirement for Rad59 when DNA ligase I is limiting in yeast. The function of Rad59 seems to be independent of Slx4 and Rad1/Rad10 and therefore unrelated to the SSA pathway.

### Deletion of *RAD59* Resulted in an Increase of Stalled Replication Forks in *cdc9* Mutants

To better understand the role of Rad59 in DNA ligase I deficiency, we characterized *cdc9-td rad59Δ* mutants more rigorously. The cells grew similar to wild-type and *rad59Δ* mutants on glucose plates at 28°C (Figures 6A and S5). In contrast, when they were spotted on 2% galactose plates, growth was inhibited, even when additional copper was added to express Cdc9 (Figure 6A). The phenotype was further exacerbated at 37°C, which facilitated degradation of Cdc9-td. The growth phenotype of *cdc9-td rad59Δ* mutants was thus very similar to that of *cdc9-1 rad59Δ* cells (Figures 5A and 6A).

**Figure 7:**
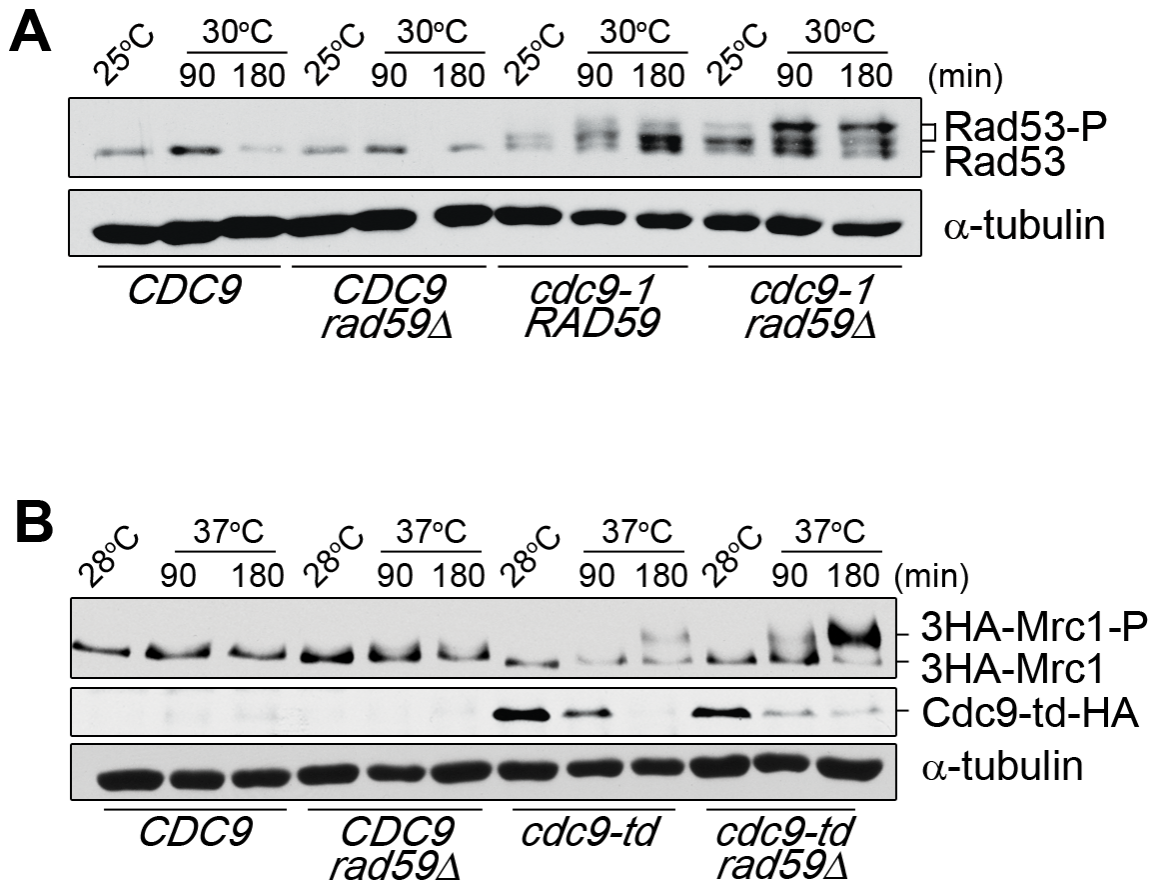
Deletion of *RAD59* in *cdc9-td* mutants displayed an increase in Mrc1 phosphorylation. (**A**) Asynchronous cultures of the indicated strains were grown at 25°C and subsequently shifted to 30°C for 90 or 180 min. Unmodified and phosphorylated Rad53 was detected using an anti-Rad53 antibody. (**B**) Asynchronous cultures were grown at 28°C and subsequently shifted to 37°C for the indicated time. Unmodified and phosphorylated 3HA-Mrc1 and Cdc9-td-HA levels were monitored using an anti-HA antibody. α-tubulin was used as a loading control.

Besides its well-documented role in break-induced replication [40], [41], Rad59 has recently been implicated to function at stalled replication forks to promote spontaneous recombination between inverted repeats [38]. Furthermore, a different study in *S. pombe* provided evidence that Rad52 could associate with stalled replication forks independently of Rad51 [42]. Since Rad52 forms distinct complexes with either Rad51 or Rad59, we postulated that Rad52/Rad59 might be active at stalled replication forks in *cdc9* mutants. Because PCNA^K107-Ub^ likely resides at stalled forks [16], we first determined whether deletion of *RAD59* had any effect on the status of PCNA ubiquitination. When we shifted both *cdc9-1 rad59Δ* and *cdc9-td rad59Δ* to their non-permissive temperatures, PCNA mono-ubiquitination at K107 remained intact (Figure 6B). These data suggested that *RAD59* functions either downstream of PCNA^K107-Ub^ or in a parallel pathway. If *RAD59* were to function downstream of PCNA ubiquitination to promote Rad53 checkpoint activation, we expected *cdc9-td rad59Δ* double mutants to exhibit reduced Rad53 activation. To determine whether Mrc1-mediated activation of Rad53 is reduced in these mutants, we examined the phosphorylation status of both Mrc1 and Rad53 [15]. To our surprise, we observed an increase in Mrc1 and Rad53 hyper-phosphorylation in *cdc9-td rad59Δ* as compared to *cdc9-td* cells (Figures 7A, B and S6). Since Mrc1 resides at replication forks to enhance Mec1 activation and promote Rad53 phosphorylation [17], these findings are consistent with the notion that Rad59 plays a role to promote replication fork progression through S phase in the absence of DNA ligase I and is required to relieve Rad53 activation.

## Discussion

In this study, we establish that nicked replication intermediates caused by defects in DNA ligase I trigger PCNA mono- and poly-ubiquitination independently of K164 (Figure 1). In support of this notion, expression of either *S. cerevisiae* wild-type Cdc9 or *Chlorella* virus DNA ligase was able to complement *cdc9-1* cell viability and revert PCNA ubiquitination (Figures 1 and 2). A previous study in *cdc9* mutants demonstrated that Okazaki fragments accumulate as ligatable nicks that can be readily joined upon re-induction of DNA ligase I expression [11]. Moreover, despite the failure to ligate Okazaki fragments, replicated DNA is still assembled into nucleosomes [43], and DNA ligase I is active on nucleosomal substrates *in vitro* [44]. This would suggest that cells can recognize both “clean” (3′-OH and 5′-PO_4_) and “dirty” (3′-OH and 5′-AMP) nicks in the context of chromatin. It is likely that limited amounts of DNA ligase I cause abortive ligation reactions leaving behind adenylated nicks. This idea is also consistent with reports demonstrating the presence of both types of nicks in human 46BR.1G1 DNA ligase I-deficient cells [20]. The data presented in this study lead us to propose that both types of nicks trigger PCNA ubiquitination, at least in budding yeast. Since this lower eukaryote lacks a homolog of the mammalian nick sensor poly (ADP-ribose) polymerase-1 (PARP-1) [45], we further postulate that PCNA ubiquitination serves as an integral part of a nick-sensory pathway during DNA replication. In agreement with previous data indicating that PCNA^K107-Ub^ was necessary for robust Rad53 hyper-phosphorylation, we demonstrate here that inactivation of Rad53 by mutating PCNA-K107 in *cdc9* mutants or by overexpressing a dominant-negative Rad53 kinase-dead mutant (Rad53-K221A/D339A) [28], [29] fails to arrest cells in S phase (Figure 3). Altogether, we conclude that budding yeast can recognize the accumulation of unligated Okazaki fragments and triggers PCNA ubiquitination at K107, not K164. Moreover, ubiquitination at K107, which is positioned within the so-called loop J of PCNA [46], is a prerequisite to induce Rad53-mediated delay of S phase progression [16].

**Figure 8:**
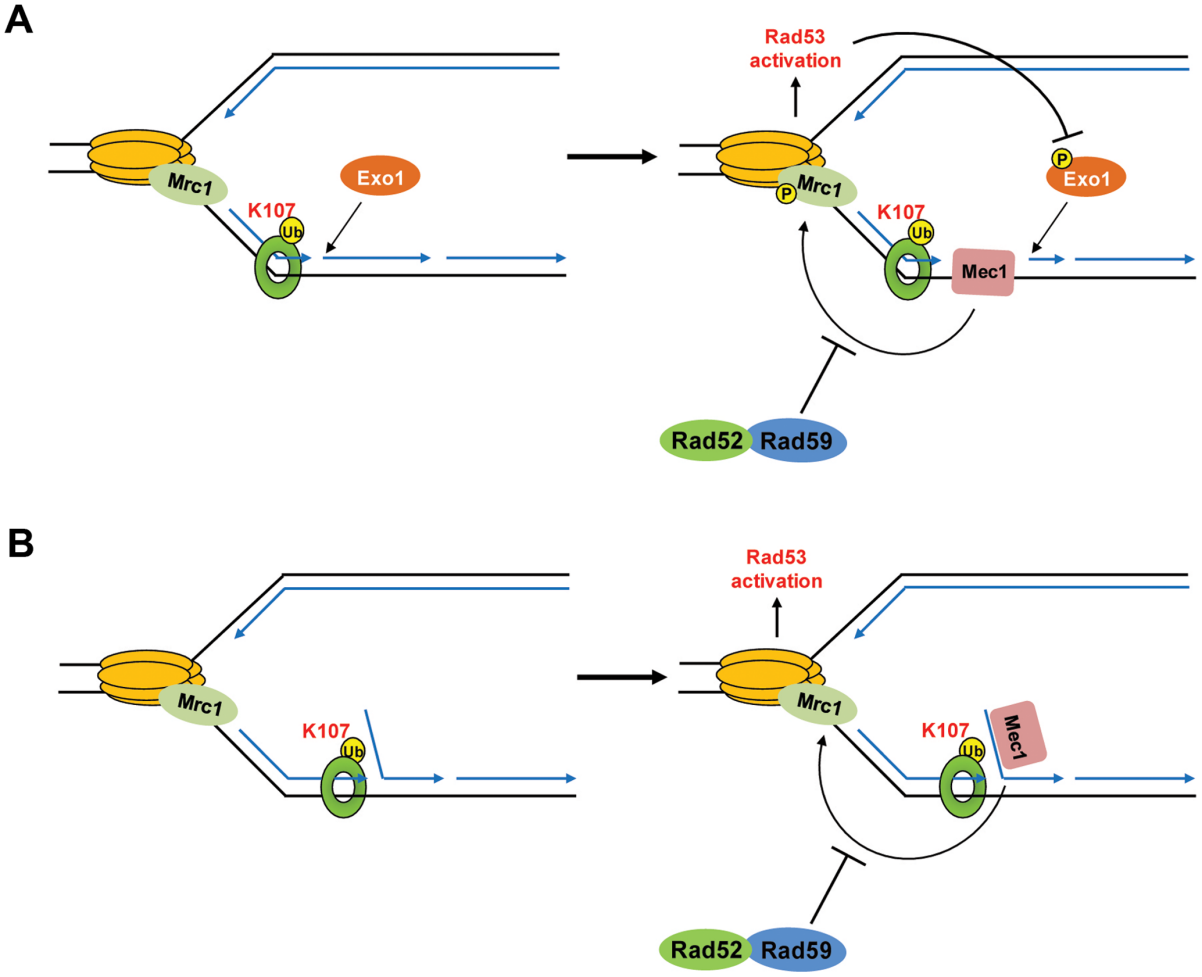
Hypothetical models to explain how Rad53 might be regulated at PCNA^K107-Ub^-flagged replication forks. (**A**) In the absence of Cdc9 activity, we suggest that PCNA ubiquitination, in conjunction with Exo1, promotes the generation of ssDNA on the lagging strand template (left). ssDNA regions recruit Mec1 to the stalled replication fork. Mec1 then phosphorylates Mrc1 at the fork, which leads to Rad53 hyper-phosphorylation to delay cells in S phase (right). Rad53, in turn, has been shown to phosphorylate Exo1 to inhibit further nascent strand degradation [62]. (**B**) Alternatively, pol-δ may displace the downstream Okazaki fragment, generating a 5′-flap (left). The 5′-flap may bind to RPA (not shown), thereby recruiting Mec1 to chromatin in order to phosphorylate Mrc1 at the fork. In both, **A** and **B**, Rad59 acts to suppress Mrc1 phosphorylation at the fork. As described in more detail in the text, Rad59 acts in concert with Rad52.

How PCNA^K107-Ub^ promotes S phase checkpoint activation is unclear. Initiating the S phase checkpoint cascade requires replication protein A (RPA) coated ssDNA (ssDNA-RPA) to recruit Mec1/Ddc2 [47]. Thus, some processing of the nicks is necessary to generate such ssDNA-RPA structures. It is possible that PCNA ubiquitination facilitates this particular step. The redundant roles of Exo1 and Xrs2 (X-ray sensitive 2, a yeast homolog of human NBS1), a component of the MRX complex, have been implicated in the degradation of nascent DNA in response to stalled replication forks [48]. We speculate that they may also facilitate processing in the context of PCNA ubiquitination. Unlike the deletion of MRX components, which is lethal in *cdc9* mutants (Table S3; [34]), deletion of *EXO1* in *cdc9-1* cells yielded viable double mutants that displayed a 5–10 fold increase in temperature sensitivity (Figure S7A). This result suggested that Exo1 plays some role in *cdc9-1* cell viability. However, Exo1 did not appear to be crucial for PCNA ubiquitination nor for Rad53 activation in *cdc9-1* mutants (Figures S7B and C). At this point we consider it highly likely that multiple different exonuclease activities contribute to the conversion of nicks into ssDNA-RPA structures, and that Exo1 might be one of them (Figure 8A). In an alternative model, which is not mutually exclusive to the events proposed above, unligated Okazaki fragments could form flaps, which are subsequently bound by RPA and recruit Mec1 (Figure 8B). There is recent precedence for such a scenario in *dna2* mutants, which accumulate long flaps at the 5′-termini of unprocessed Okazaki fragments [49]. We speculate that PCNA^K107-Ub^ may actively facilitate 5′-flap formation, thereby enabling Mec1 phosphorylation (Figure 8B). Consistent with this model, mono-ubiquitinated PCNA still supports DNA synthesis by pol-δ, but prevents Fen1 from accessing 5′-flaps *in vitro* [50]. This would explain why 5′-flaps may have an extended half-life and could serve as a “docking” platform for Mec1, as long as they are not processed by Dna2 (Figure 8B). Moreover, this model would provide a rationale for the role of Rad59 in checkpoint suppression. We propose here that Rad59 is required for the re-annealing of these flaps, which promotes replication fork progression by deactivating Mec1 (Figure 8B). Although Rad59 is unable to anneal RPA-coated ssDNA, it has been shown to enhance Rad52-mediated annealing in the presence of RPA and salt conditions that destabilize Rad52-ssDNA complexes [51]. Moreover, Rad52 and Rad59 interact *in vivo*, suggesting strongly that if Rad59 has a role in the re-annealing of flaps, it executes this function in the context of Rad52. This re-annealing function would also prevent the two nascent strands from annealing to each other, leading to the formation of a double-stranded end, a potential substrate for Exo1. Thus, Rad59 may not just suppress checkpoint activation, but also fork regression and subsequent DNA resection.

### How do Cells Cope with Accumulated Nicks in *cdc9* Mutants?

Since Cdc9 is the only ligase that can seal nicks during lagging strand synthesis, it is unclear what mechanisms are in place to “resolve” persistent nicks in the presence of limited DNA ligase I activity. In *cdc9-1* cells, the observation that Mrc1 and Rad9 are crucial for S phase delay suggests the presence of both ssDNA at stalled replication forks and DSBs, respectively [16]. It is conceivable that DSBs are either ligated by Dnl4 or must await Mec1 inactivation, as Mec1 activity inhibits HR-mediated DSB repair during S phase [52], [53]. Rad59 appears to have an important role in aiding Mec1 deactivation by suppressing Mrc1 phosphorylation (Figure 7B). Mrc1 is phosphorylated at stalled replication forks and the proximity of Mec1 to phosphorylated Mrc1 is required for enhancing Mec1 activation [17]. Therefore, suppression of Mrc1 phosphorylation will counteract this process and provides one explanation for the observation that Rad59 promotes *cdc9-1* survival (Figures 8A and B). How Rad59 suppresses Mrc1 phosphorylation on the molecular level and facilitates replication fork progression is not clear. As mentioned above, it is possible that Rad52-Rad59 dimers simply promote the re-annealing of detached flaps that might form at the ends of the nicked replication intermediates. These flaps would likely interfere with proper nucleosome formation behind the fork, which in turn, slows down fork progression [54]. This might in fact be beneficial for *cdc9* mutants, as it provides the cells with extra time to recycle the little DNA ligase I activity that they have at their disposal. We like this idea because the deletion of each of three individual genes, *RLF2*, *MSI1* and *ITC1*, which comprise subunits of the chromosome assembly factor 1 [55], improve *cdc9-1* viability (Table S3). However, since replication fork slow down generally increases the risk of replication fork collapse, the cell likely has means to counterbalance this effect. We envision that Rad59 could provide such counterbalance by preserving the structure of the ligatable nicks, thereby promoting nucleosome assembly and ultimately replication fork progression. Because deletion of *RAD59* exhibited an increase in Mrc1 phosphorylation in *cdc9* mutants, Rad59 is required for suppression of replication fork stalling. This is a novel role that has not been reported previously. Whether Rad59 functions in the same pathway as PCNA^K107-Ub^ is unclear. We exclude the possibility that Rad59 promotes Rad53 activation downstream of PCNA ubiquitination. However, it is possible that Rad59 is recruited by ubiquitinated PCNA, either directly or indirectly, to help alleviate fork arrest.

It is also worthwhile to note that there is a limited set of only 23 mutants for which synthetic sickness/lethality with *rad59Δ* has been described [56]–[58] (Table S2). Intriguingly, among those are four other lagging strand specific mutants, *pol3-13*, defective in the catalytic subunit of the replicative pol-δ, *pol32Δ*, defective in a subunit of pol-δ, *rad27Δ*, defective in flap endonuclease, and *dna2-1* defective in the endonuclease/helicase implicated in flap processing [56]–[59]. This suggests that *RAD59* may play a general role at stalled forks in response to defects in Okazaki fragment maturation. Thus, the molecular function of *RAD59* in *pol3-13*, *pol32Δ, rad27Δ* and *dna2-1* cells needs to be further investigated in the future. We speculate that Rad59 might have a similar role in *dna2-1* mutants as described here for DNA ligase I-deficient cells in suppressing Mrc1 activation.

In summary, we present evidence that eukaryotic cells utilize PCNA ubiquitination as a means to monitor the status of Okazaki fragment maturation. We envision that this system serves as a local marking device to single out forks that display delayed joining of Okazaki fragments. Although we have yet to uncover all of the molecular pieces that help to translate the accumulation of nicked DNA into a robust S phase checkpoint response, our results demonstrate that active mechanisms are in place to suppress fork stalling in response to problems that cannot be sensed by stalled polymerases, but rather arise during lagging strand processing.

## Materials and Methods

### Yeast Strains

Yeast strains used in this study are isogenic derivatives of SSL204, YKL83, or the RDKY3615 and the relevant genotypes are shown in Table S1. Various genes (*RAD1, RAD2, RAD10, RAD14, RAD59,* and *SLX4*) were deleted using a one-step PCR gene replacement method [60]. Gene disruption was confirmed by sequencing. PCNA lysine mutants were generated as described [16].

To construct the N-terminally tagged hemagglutinin (HA) *MRC1* (*3HA-MRC1*) in the endogenous locus, two-step PCR-mediated integration was performed as described [61]. Briefly, two pairs of oligonucleotides were synthesized to amplify *3HA-MRC1* and the *KanMX4* marker on two separate, but overlapping fragments. The *3HA-MRC1*, which includes 39 bp upstream and 288 bp downstream sequence from its start and stop codons, respectively, was amplified from pRS405-*3HA-MRC1* (a gift from DM Koepp, University of Minnesota) using 5′-CGTTATTCGCTTTTGAACTTATCACC-3′ and 5′-*GGGATCCGTCGACCTGCAGCGTACG* GCAAGATGCTTTGAATACAGAACTG-3′. The resulting PCR product contains a 25 bp overlapping segment with the *kanMX4* cassette at the 5′-end (italicized sequence). The *KanMX4* gene was amplified using 5′-CGTACGCTGCAGGTCGACGGATCC C-3′ and 5′-AGCTTCTGGAGTTCAATCAACTTCTTCGGAAAAGATAAAAAACCAATCGATGAATT CGAGCTCGTTTTCG-3′ to create a fragment that overlapped with 40 bp immediately downstream of the endogenous *MRC1* locus (underlined sequence). PCR products were combined, denatured at 94°C for 3 to 4 min, cooled to room temperature and transformed into the desired yeast strains.

### Plasmids

The *CDC9* gene with its endogenous promoter was initially cloned into the vector pRS313 using the *BamH*I restriction site (*pCDC9*, a gift from DM Livingston, University of Minnesota). p*cdc9-NΔ60*, p*cdc9-K419A*, and p*cdc9-K598A* plasmids were derived from the plasmid p*CDC9* using Quikchange Lightning Site-Directed Mutagenesis (Agilent, Santa Clara, CA). p*ChVLig-3HA* was constructed by cloning two different PCR fragments into the vector pRS313. Between the *BamH*I-*Xho*I sites of the p*ChVLig-3HA* plasmid is the *CDC9* promoter (*CDC9pro*, 449-bp) driving the expression of the *Chlorella* virus DNA ligase coding sequence (*ChVLig*, a gift from S Shuman, Memorial Sloan Kettering Institute) that is followed by three HA tags at its C-terminus (*BamH*I-*CDC9pro*-*Cla*I-*ChVLig-3HA*-*Xho*I) [9]. To overexpress *Chlorella* virus DNA ligase, the *ChVLig-3HA* fragment was subcloned from the p*ChVLig*-*3HA* plasmid into the pRS423gal vector (p*gal*) under the control of the *GAL10* promoter using *Cla*I-*Xho*I sites (p*gal*-*ChVLig-3HA*). All constructs were confirmed by DNA sequence analysis.

### Synthetic Genetic Array (SGA) Analysis

A genome-wide screen for *CDC9* genetic interactions was conducted as described [30]. Briefly, a *cdc9-1* mutant strain marked with a nourseothricin (*NatMX4*) resistance cassette and harboring the SGA haploid specific markers and reporter [30] was mated to an array of ∼4000 viable *S. cerevisiae* deletion mutants. Nourseothricin- and geneticin-resistant heterozygous diploid mutants were selected and sporulated and *MATa cdc9-1* double mutants were subsequently selected as described [30].

To confirm the SGA results, all gene deletions were constructed in a SSL204 *MATa* strain and crossed with an isogenic *cdc9-1 MATα* strain. Diploid cells were sporulated at 25°C and dissected. Plates were incubated for 3–5 days at either 25°C or 30°C.

### Complementation of *cdc9-1* Temperature Sensitivity

All exogenous Cdc9 plasmids and the pRS313-*ChVLig*-*3HA* plasmids were transformed into wild-type, *cdc9-1* and *cdc9-1 pol30-K164R* strains. 10-fold serial dilutions of cells were spotted on appropriate medium. Plates were incubated for 2 to 3 days at indicated temperatures. Temperature shift experiments were carried out as described [16]. Briefly, cells were grown overnight to mid-log phase (OD_600_ = 0.6) at 25°C and shifted to the restrictive temperature of 35°C for 3 hr. For degron strains, cells were grown overnight at 28°C in YP plus 2% raffinose and supplemented with 10 uM CuSO_4_ to induce gene expression. Once cells grew to mid-log phase, the cells were switched to YP plus 2% galactose without CuSO_4_ for an addition 30 min at 28°C and subsequently shifted to 37°C.

For the overexpression experiments of the ChVLig-3HA, both p*gal* and p*gal*-*ChVLIG*-*3HA* plasmids were transformed into either wild-type or *cdc9-1* strains. 10-fold serial dilutions of cells were spotted on minimal medium lacking histidine (Sc-His), but containing either 2% glucose or 2% galactose. Plates were incubated for 4 to 5 days at either 25°C or 35°C. For temperature shift experiments, asynchronous cultures were grown overnight to mid-log phase at 25°C in Sc-His medium containing 2% raffinose. Cultures were split and shifted to 35°C for 3 hr in the presence of either 2% glucose or galactose. To increase the efficiency of Cdc9-td degradation, Ubr1 was overexpressed from a galactose-inducible promoter in the presence of 2% galactose.

### Complementation of cdc9-1* K107R by *cdc9-1* Overexpression

*cdc9-1* cDNA was PCR amplified from p*cdc9-1* (a gift from DM Livingston at the University of Minnesota) with primers designed to engineer a BamHI restriction site at the 5′ end of the fragment and a HindIII site at the 3′ end. The resulting fragment was digested and ligated into the pBM272 vector under control of the *GAL1,10* promoter. The identity of the resulting p*gal-cdc9-1* construct was confirmed by sequencing. p*gal-cdc9-1* and p*gal* empty vector were transformed into *cdc9-1* and cdc9-1* K107R strains at 25°C. 5-fold serial dilutions of the resulting transformants were spotted on minimal medium lacking uracil (Sc-Ura) and containing either 2% glucose or 2% galactose. Plates were incubated at 33°C or 35°C for 5 days.

### Characterization of GCR and *CAN1* Forward Mutation Rates

GCR rates and *CAN1* forward mutation rates were determined as described [32], [33]. Briefly, the GCR or mutation rates from two independent isolates were determined by fluctuation analyses twice using the method of the median. Each experiment was performed using 11 cultures and the average value from two different clones is reported.

### Sensitivity to UV, MMS and γ-irradiation

Overnight cultures of the indicated strains were serially diluted and spotted onto YPD plates or YPD plates containing MMS. For UV- or γ-irradiation, strains were spotted onto YPD plates and irradiated as indicated. Plates were incubated at 30°C for 2 days.

### Cell Cycle and FACS Analysis

Cell cycle progression was monitored using flow cytometry as described [16]. DNA was stained by Sytox Green and all FACS samples were analyzed using a Becton Dickinson FACS Calibur.

### Protein Preparation and Western Blot Analysis

Total protein extracts were prepared from cycling cells using TCA precipitation and protein expression was analyzed by immunoblotting [16]. Endogenous Cdc9 and Rad53 proteins were detected using anti-Cdc9 (1∶12000, a gift from AE Tomkinson, University of New Mexico) and anti-Rad53 (1∶1000, a gift from JF Diffley, Cancer Research UK London Research Institute) antibodies, respectively. All HA-tagged proteins were detected using either an anti-HA (1∶3000, 16B12, Covance) or anti-HA-HRP conjugated (1∶250, 3F10, Roche) antibody, respectively. Both unmodified and ubiquitinated forms of PCNA were detected using an anti-yeast PCNA antibody as described (1∶4000, clone S871, a gift from Z Zhang, Mayo Clinic, MN and BW Stillman, Cold Spring Harbor Laboratory, NY) [16]. For detection of mono-ubiquitinated PCNA, protein extracts were diluted prior to fractionating by SDS-PAGE gel electrophoresis as described [16]. α-tubulin served as a loading control.

## Supporting Information

Figure S1 **Overexpression of** ***cdc9-1*** **rescues growth sensitivity of** cdc9-1* K107R. Successive 5-fold dilutions of *cdc9-1* and cdc9-1* K107R carrying *pgal* empty vector or *pgal-cdc9-1* were spotted on minimal medium lacking uracil and containing either 2% glucose or 2% galactose. Overexpression of *cdc9-1* from the *pgal-cdc9-1* plasmid was under the control of the *GAL1,10* promoter. Plates were incubated at 33°C and 35°C for 5 days.
(TIF)

Figure S2 **Differential DNA damage sensitivity and canavanine resistance of various PCNA mutants.** (**A**) Successive 10-fold dilutions of either wild-type or different PCNA lysine to arginine mutants were spotted on rich medium and treated with different DNA damaging agents as indicated. The *mec1Δ sml1Δ* strain was used as a negative control. (**B**) *CAN1* forward mutation rates of two independent isolates of different PCNA mutants are shown.
(TIF)

Figure S3 ***cdc9-1*** **mutants exhibit enhanced gross chromosomal rearrangements.** Gross chromosomal rearrangement (GCR) rates of wild-type and *cdc9-1* cells were analyzed as described [32], [33]. GCR rates from two independent isolates were determined by fluctuation analyses twice using the method of the median. Each experiment was performed using 11 cultures and the average value from two different clones is reported.
(TIF)

Figure S4 ***RAD51/RAD52*** **-mediated homologous recombination is required for** ***cdc9-1*** **survival.** Diploid strains were dissected and incubated at 25°C. All haploid genotypes are as indicated. Four independent tetrads (1–4) are laid out horizontally. (**A**) Segregates from *CDC9/cdc9-1 rad51Δ/RAD51* diploids. (**B**) Segregates from *CDC9/cdc9-1 rad52Δ/RAD52* diploids.
(TIF)

Figure S5 ***RAD1*** **and** ***RAD59*** **work in separate pathways in** ***cdc9-td*** **mutants.** (**A**) Successive 10-fold dilutions of the indicated strains were grown on YP plates containing either 2% glucose or 2% galactose at 28°C and 37°C. (**B**) Successive 5-fold dilutions of the indicated strains were grown on YP plates containing either 2% glucose or 2% galactose at 28°C and 37°C. Galactose induces the expression of *UBR1*, which promotes degradation of the heat-inducible degron fusion protein, Cdc9-td.
(TIF)

Figure S6 **Deletion of** ***RAD59*** **in** ***cdc9-td*** **mutants causes an increase in Rad53 phosphorylation.** Asynchronous cultures were grown at 28°C and subsequently shifted to 37°C for 1.5 and 3 hr. Cdc9-td-HA and Rad53 was detected using anti-HA-HRP and anti-Rad53 antibodies, respectively. The asterisk indicates a non-specific band that runs on top of the band for hyper-phosphorylated Rad53 in this strain background.
(TIF)

Figure S7 **Deletion of** ***EXO1*** **does not alter PCNA mono-ubiquitination and Rad53 phosphorylation in** ***cdc9-1*** **mutants.** (**A**) Successive 10-fold dilutions of the indicated strains were spotted on YPD plates and incubated for 3 days at the indicated temperatures. (**B, C**) Strains shown in **A** were grown asynchronously to mid-log phase at 25°C and subsequently shifted to the indicated temperature for 1.5 and 3 hr. PCNA and its ubiquitinated forms and Rad53 were detected with anti-PCNA (S871) and anti-Rad53 antibodies, respectively. α-tubulin served as a loading control.
(TIF)

Table S1 **List of yeast strains used in this study.**
(DOCX)

Table S2 **Complete profiles of indicated query genes.** Genetic interactions involving the *pol32Δ*, *rad27Δ*, and *rad6Δ* mutants were obtained from published studies [56]. Genetic interactions involving all other deletion mutants and temperature-sensitive mutants were obtained from the most recent SGA dataset (C. Boone, unpublished data, 2 January 2012). Both of these sources use SGA technology to compare query mutants to a collection of 4000 deletion mutants. PH designates alleles that came from Phil Hieter [63], [64]. All genetic interactions were scored as described [31].
(XLSX)

Table S3 **Genetic interactions with** ***cdc9-1*** **mutants.** Negative and positive genetic interactions with *cdc9-1* were included only if the epsilon scores were either below −0.09 or larger than 0.09 and p-values were below 0.15.
(XLSX)
