# Supplementary figures and images for: Unligated Okazaki Fragments Induce PCNA Ubiquitination and a Requirement for Rad59-Dependent Replication Fork Progression
Source: PLoS One. 2013 Jun 18;8(6):e66379. doi: 10.1371/journal.pone.0066379 (PMC3688925; doi:10.1371/journal.pone.0066379)

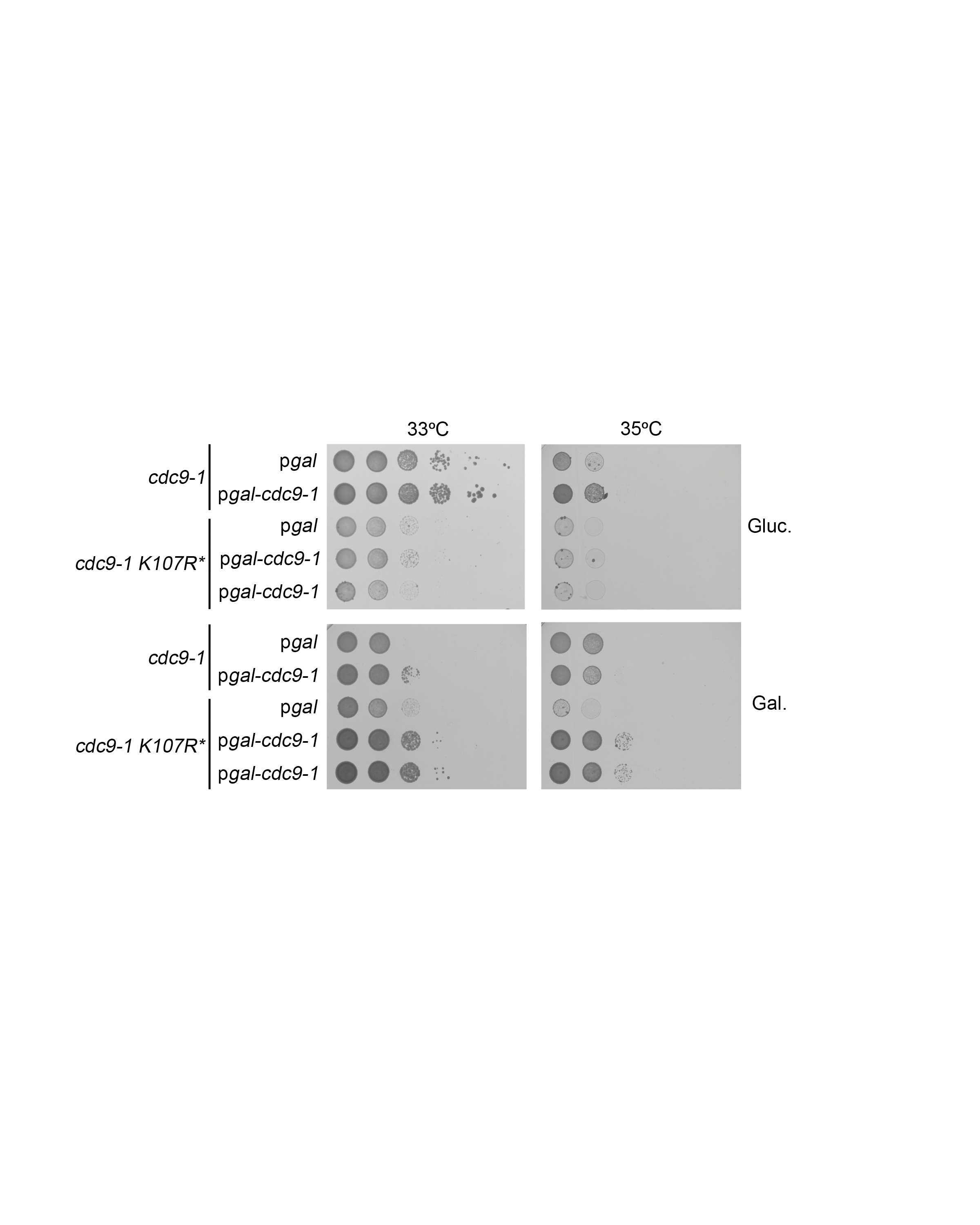

Supplement: Figure S1 — Overexpression of cdc9-1 rescues growth sensitivity of cdc9-1* K107R. Successive 5-fold dilutions of cdc9-1 and cdc9-1* K107R carrying pgal empty vector or pgal-cdc9-1 were spotted on minimal medium lacking uracil and containing either 2% glucose or 2% galactose. Overexpression of cdc9-1 from the pgal-cdc9-1 plasmid was under the control of the GAL1,10 promoter. Plates were incubated at 33°C and 35°C for 5 days. (TIF) [file pone.0066379.s001.tif]

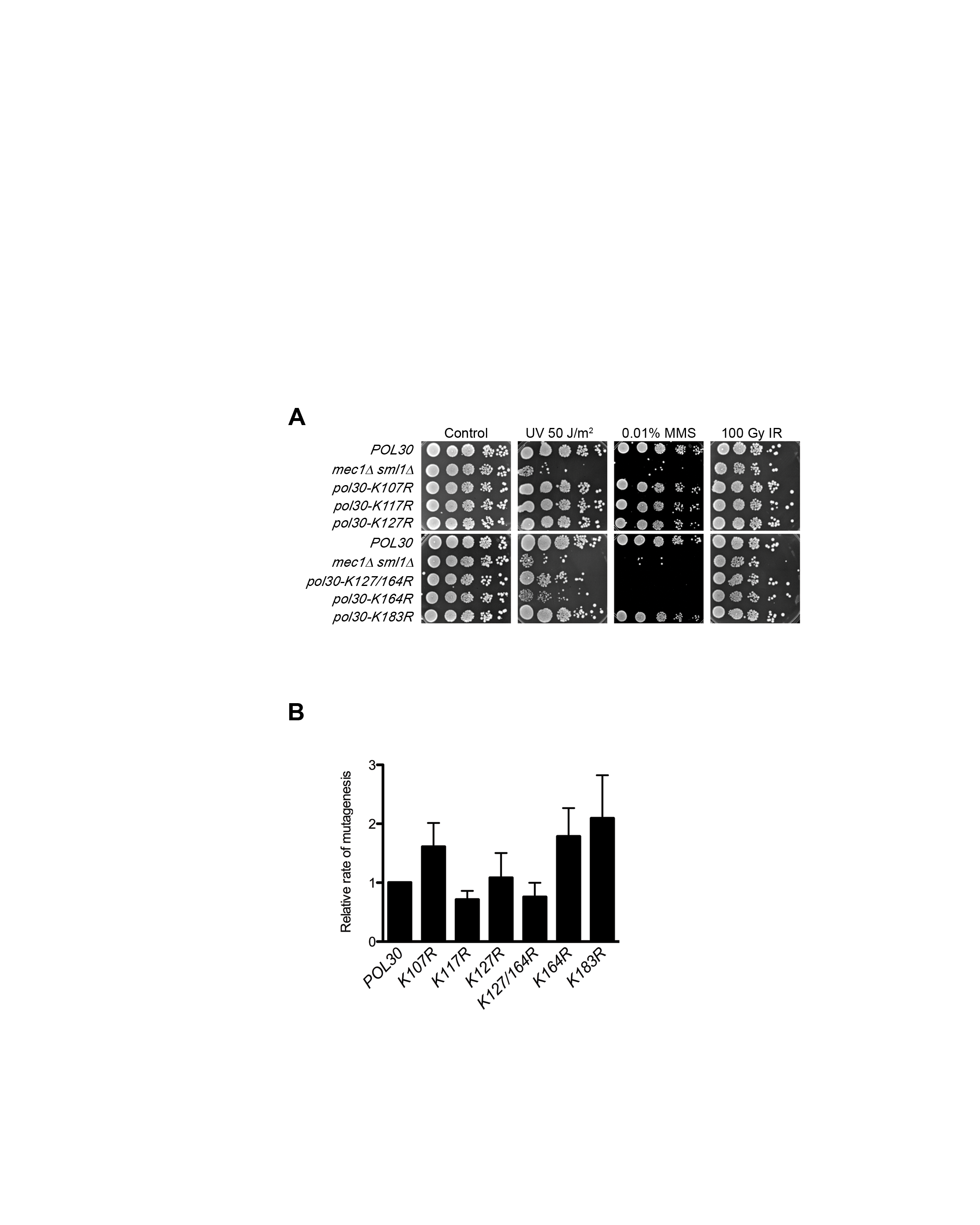

Supplement: Figure S2 — Differential DNA damage sensitivity and canavanine resistance of various PCNA mutants. (A) Successive 10-fold dilutions of either wild-type or different PCNA lysine to arginine mutants were spotted on rich medium and treated with different DNA damaging agents as indicated. The mec1Δ sml1Δ strain was used as a negative control. (B) CAN1 forward mutation rates of two independent isolates of different PCNA mutants are shown. (TIF) [file pone.0066379.s002.tif]

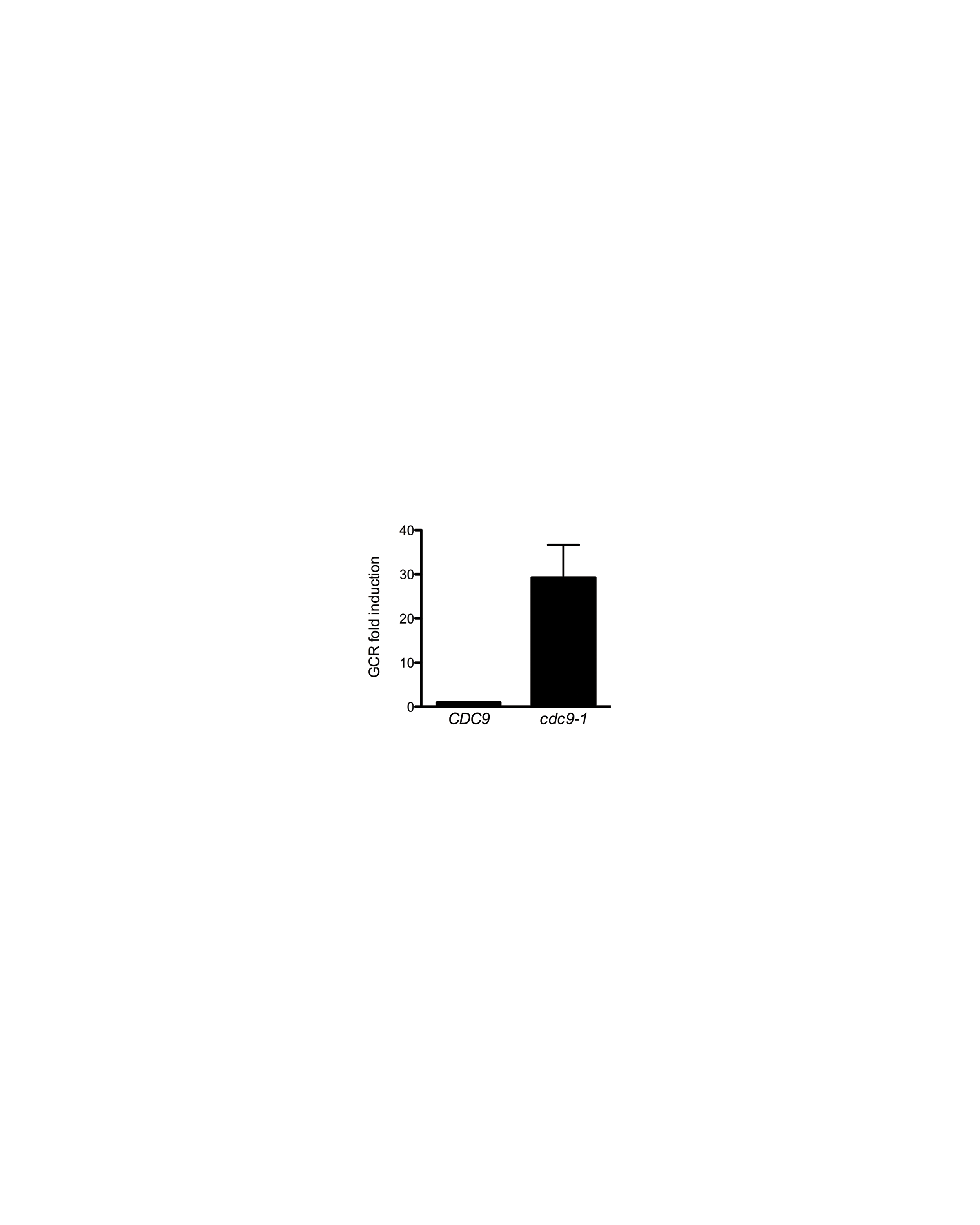

Supplement: Figure S3 — cdc9-1 mutants exhibit enhanced gross chromosomal rearrangements. Gross chromosomal rearrangement (GCR) rates of wild-type and cdc9-1 cells were analyzed as described [32], [33]. GCR rates from two independent isolates were determined by fluctuation analyses twice using the method of the median. Each experiment was performed using 11 cultures and the average value from two different clones is reported. (TIF) [file pone.0066379.s003.tif]

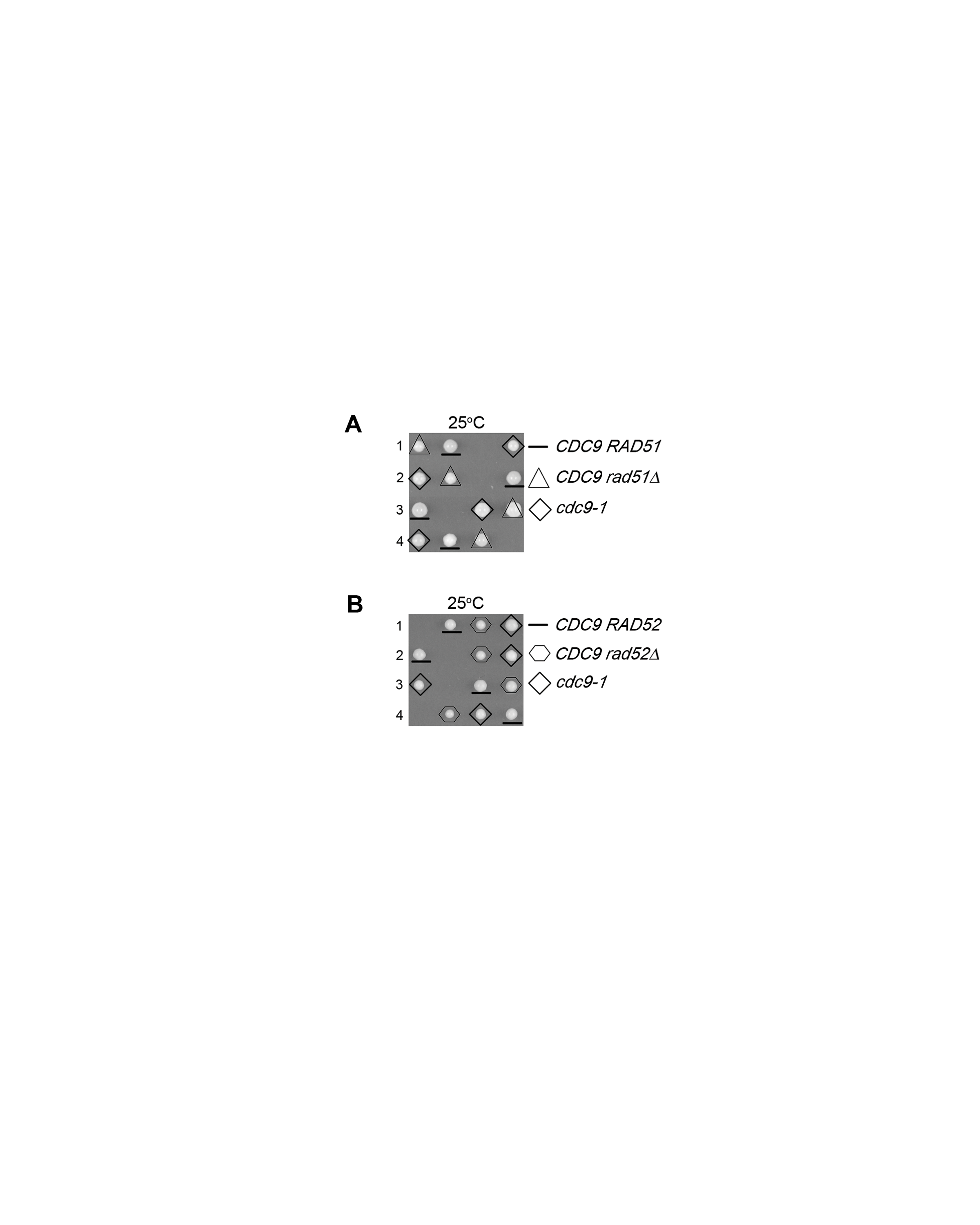

Supplement: Figure S4 — RAD51/RAD52 -mediated homologous recombination is required for cdc9-1 survival. Diploid strains were dissected and incubated at 25°C. All haploid genotypes are as indicated. Four independent tetrads (1–4) are laid out horizontally. (A) Segregates from CDC9/cdc9-1 rad51Δ/RAD51 diploids. (B) Segregates from CDC9/cdc9-1 rad52Δ/RAD52 diploids. (TIF) [file pone.0066379.s004.tif]

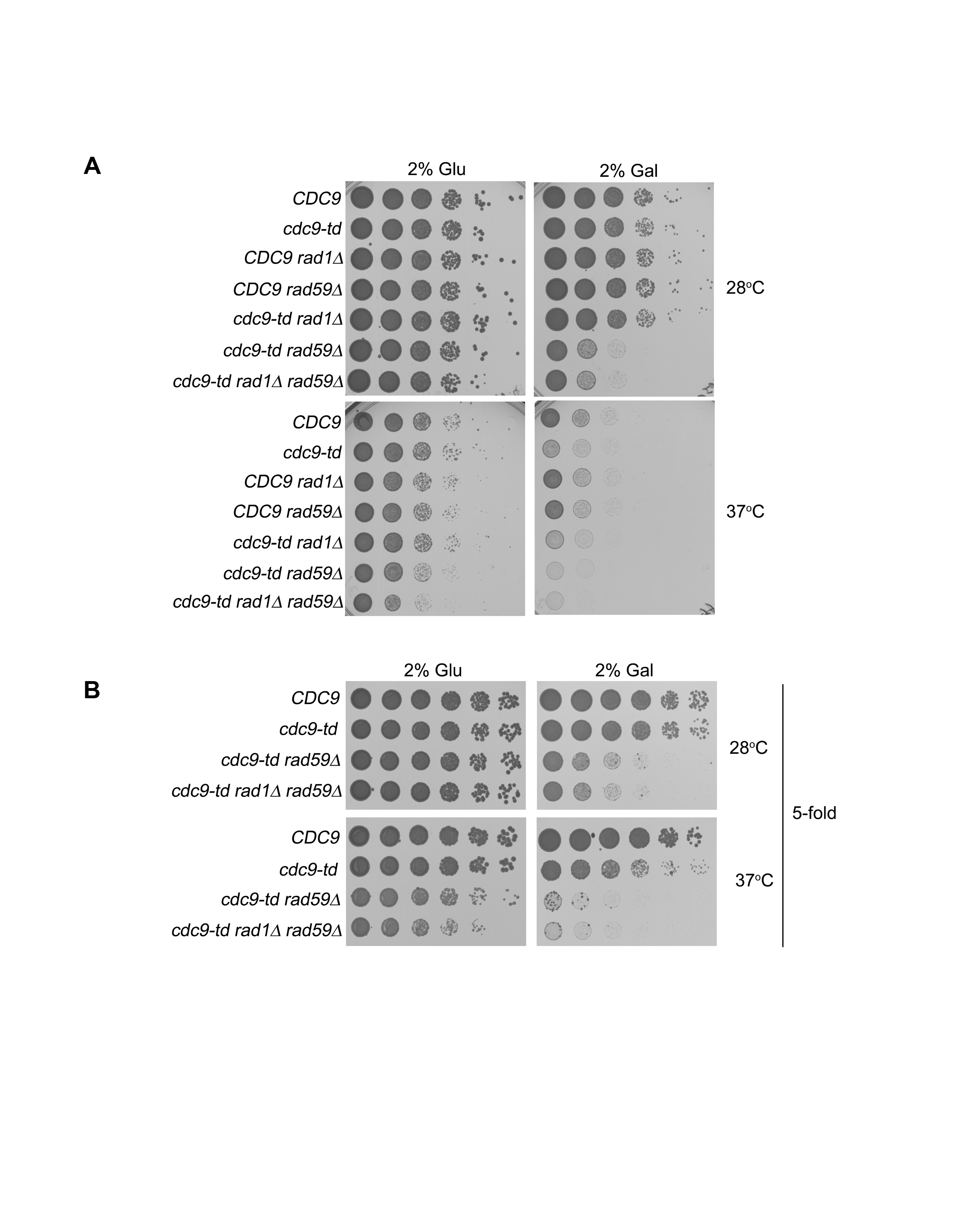

Supplement: Figure S5 — RAD1 and RAD59 work in separate pathways in cdc9-td mutants. (A) Successive 10-fold dilutions of the indicated strains were grown on YP plates containing either 2% glucose or 2% galactose at 28°C and 37°C. (B) Successive 5-fold dilutions of the indicated strains were grown on YP plates containing either 2% glucose or 2% galactose at 28°C and 37°C. Galactose induces the expression of UBR1, which promotes degradation of the heat-inducible degron fusion protein, Cdc9-td. (TIF) [file pone.0066379.s005.tif]

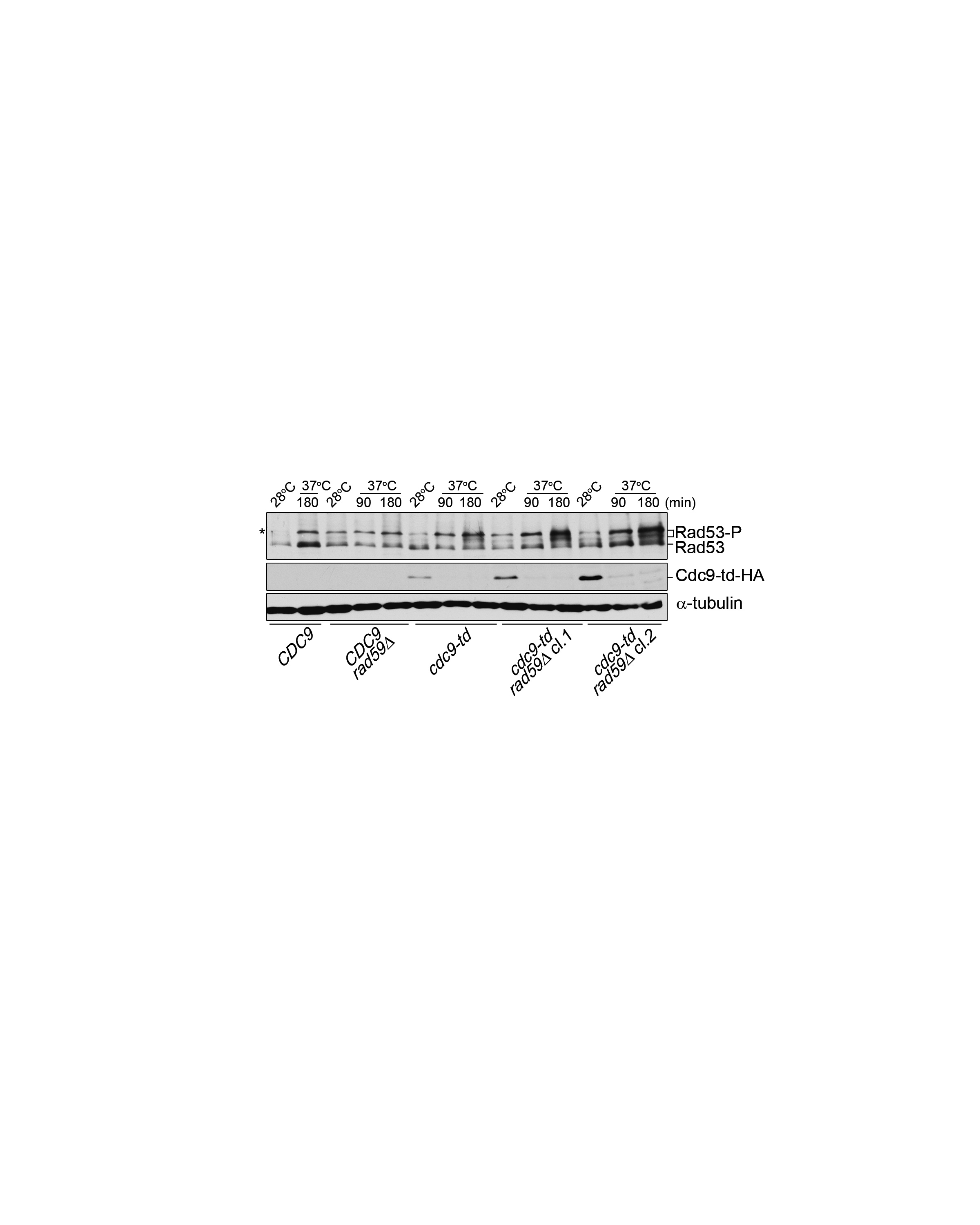

Supplement: Figure S6 — Deletion of RAD59 in cdc9-td mutants causes an increase in Rad53 phosphorylation. Asynchronous cultures were grown at 28°C and subsequently shifted to 37°C for 1.5 and 3 hr. Cdc9-td-HA and Rad53 was detected using anti-HA-HRP and anti-Rad53 antibodies, respectively. The asterisk indicates a non-specific band that runs on top of the band for hyper-phosphorylated Rad53 in this strain background. (TIF) [file pone.0066379.s006.tif]

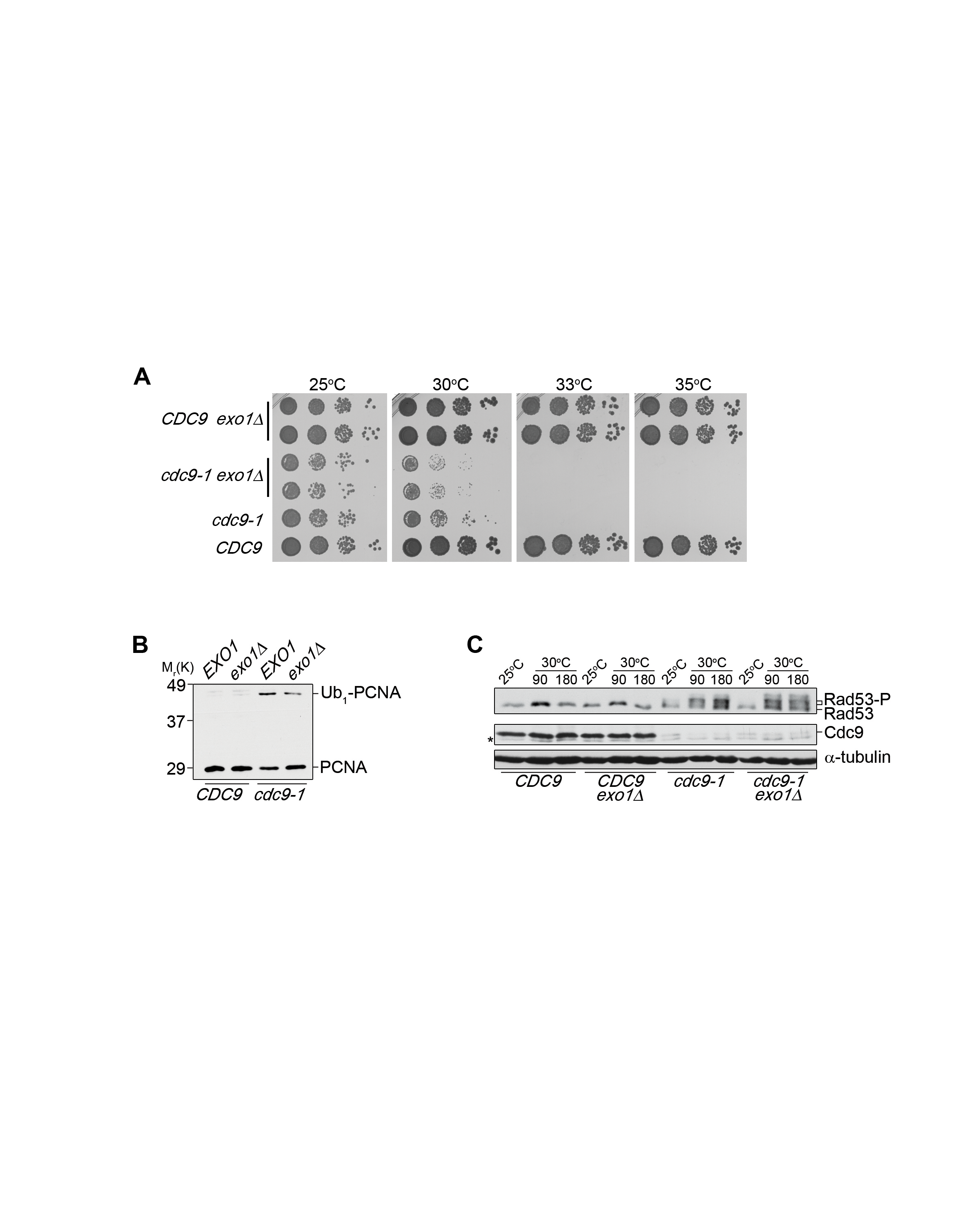

Supplement: Figure S7 — Deletion of EXO1 does not alter PCNA mono-ubiquitination and Rad53 phosphorylation in cdc9-1 mutants. (A) Successive 10-fold dilutions of the indicated strains were spotted on YPD plates and incubated for 3 days at the indicated temperatures. (B, C) Strains shown in A were grown asynchronously to mid-log phase at 25°C and subsequently shifted to the indicated temperature for 1.5 and 3 hr. PCNA and its ubiquitinated forms and Rad53 were detected with anti-PCNA (S871) and anti-Rad53 antibodies, respectively. α-tubulin served as a loading control. (TIF) [file pone.0066379.s007.tif]
