# Supplementary material for: Unligated Okazaki Fragments Induce PCNA Ubiquitination and a Requirement for Rad59-Dependent Replication Fork Progression
Source: PLoS One. 2013 Jun 18;8(6):e66379. doi: 10.1371/journal.pone.0066379 (PMC3688925; doi:10.1371/journal.pone.0066379)
Supplement: Table S1 — List of yeast strains used in this study. (DOCX) [file pone.0066379.s008.docx]

**Unligated Okazaki fragments induce PCNA ubiquitination and a requirement for Rad59-dependent replication fork progression**

Hai Dang Nguyen^1^, Jordan Becker^1, #^, Yee Mon Thu^1,#^, Michael Costanzo^2^, Elizabeth N Koch^4^, Stephanie Smith^5^, Kyungjae Myung^5^, Chad L Myers^4^, Charles Boone^2,3^, and Anja-Katrin Bielinsky^1,*^

**Table S1. List of yeast strains.**

| **Strain Name** | **Relevant Genotype** | **Source** |
| --- | --- | --- |
|  | **W303-derived strains** |  |
| YKL83 | *GAL-UBR1 (HIS3)* | [1] |
| ABy010 | *GAL-UBR1 (HIS3) bar1::LEU2* | This Study |
| ABy1643 | *GAL-UBR1 (HIS3) bar1::LEU2 MRC1::3HA-MRC1(KanMX4)* | This Study |
| ABy1596 | *GAL-UBR1 (HIS3) rad59::TRP1* Cl.1 | This Study |
| ABy1597  ABy1904 | *GAL-UBR1 (HIS3) rad59::TRP1* Cl.2  *GAL-UBR1 (HIS3) bar1::LEU2 rad1::NatR* | This Study  This Study |
| ABy1654 | *GAL-UBR1 (HIS3) bar1::LEU2 MRC1::3HA-MRC1(KanMX4) rad59::TRP1* | This Study |
| ABy008  ABy1906 | *GAL-UBR1 (HIS3) bar1::LEU2 cdc9::cdc9-td (URA3)*  *GAL-UBR1 (HIS3) bar1::LEU2 cdc9::cdc9-td (URA3) rad1::NatR* | This Study  This Study |
| ABy1541 | *GAL-UBR1 (HIS3) bar1::LEU2 cdc9::cdc9-td (URA3) MRC1::3HA-MRC1(KanMX4)* | This Study |
| ABy1598 | *GAL-UBR1 (HIS3) bar1::LEU2 cdc9::cdc9-td (URA3) rad59::TRP1* Cl.1 | This Study |
| ABy1599  ABy1908 | *GAL-UBR1 (HIS3) bar1::LEU2 cdc9::cdc9-td (URA3) rad59::TRP1* Cl.2  *GAL-UBR1 (HIS3) bar1::LEU2 cdc9::cdc9-td (URA3) rad59::TRP1* Cl.1 *rad1::NatR* | This Study  This Study |
| ABy1656 | *GAL-UBR1 (HIS3) bar1::LEU2 cdc9::cdc9-td (URA3) MRC1::3HA-MRC1(KanMX4) rad59::TRP1* | This Study |
|  | **SSL204-derived strains** |  |
| SSL204 | *MAT****a*** *ade2 his3Δ200 trp1 leu2 ura3-52* | [2] |
| ABy1321  ABy1889 | *rad1::URA3*  *rad2::URA3* | This study  This study |
| ABy1323 | *rad10::URA3* | This study |
| ABy1325 | *rad14::URA3* | This study |
| ABy1451 | *slx4::URA3* | This study |
| ABy1407 | *rad59::URA3* | This study |
| ABy1430 | *rad51::URA3* | This study |
| ABy1537 | *exo1::URA3* Cl.1 | This study |
| ABy1538 | *exo1::URA3* Cl.2 | This study |
| ABy805 | *hxt13::URA3* | This study |
| SSL212A | *rad52Δ HS::LEU2* | [2] |
| ABy1055 | pRS313 | This study |
| ABy1056 | pRS313*-CDC9* | This study |
| ABy1127 | pRS313*-cdc9-K419A* | This study |
| ABy1177 | pRS313*-cdc9-K598A* | This study |
| ABy1086 | pRS313*-cdc9-NΔ60* | This study |
| ABy1185 | pRS313*-ChVLig-3HA* | This study |
| ABy1277 | pRS423*gal* | This study |
| ABy1278 | pRS423*gal*-*ChVLig-3HA* | This study |
| ABy1146 | p*gal* | This study |
| ABy1146 | p*gal*-*rad53-K221A/D339A* (KD) | This study |
| ABy685 | *pol30::pol30K107R (LEU2)* | [3] |
| SSL612α | *cdc9-1 MAT****α*** *ade2 his3Δ200 trp1 leu2 ura3-52* | [4] |
|  | **SSL612a *(cdc9-1)*-derived strains** |  |
| SSL612a | *cdc9-1 MAT****a*** *ade2 his3Δ200 trp1 leu2 ura3-52* | [4] |
| ABy1539 | *exo1::URA3* Cl.1 | This study |
| ABy1540 | *exo1::URA3* Cl.2 | This study |
| ABy807 | *hxt13::URA3* | This study |
| ABy1057 | *cdc9-1*, pRS313 | This study |
| ABy1058 | *cdc9-1*, pRS313-*CDC9* | This study |
| ABy1128 | *cdc9-1*, pRS313-*cdc9-K419A* | This study |
| ABy1087 | *cdc9-1*, pRS313-*cdc9-K598A* | This study |
| ABy1178 | *cdc9-1*, pRS313- *cdc9-NΔ60* | This study |
| ABy1186 | *cdc9-1*, pRS313*-ChVLig-3HA* | This study |
| ABy1279 | *cdc9-1*, pRS423*gal* | This study |
| ABy1280 | *cdc9-1*, pRS423*gal*-*ChVLig-3HA* | This study |
| ABy1148 | p*gal* | This study |
| ABy1149  ABy1388-10b  ABy1890  ABy1874  ABy1391-3d | p*gal*-*rad53-K221A/D339A* (KD)  *cdc9-1 rad1::URA3*  *cdc9-1 rad2::URA3*  *cdc9-1 rad10::URA3*  *cdc9-1 rad14::URA3* | This study  This study  This study  This study  This study |
| ABy1439-4a | *cdc9-1 rad59::URA3* Cl.1 | This study |
| ABy1439-7c | *cdc9-1 rad59::URA3* Cl.2 | This study |
| ABy1439-13a  ABy1897  ABy1912  ABy1913 | *cdc9-1 rad59::URA3* Cl.3  *cdc9-1 slx4::URA3*  *cdc9-1*, pBM272-*gal*  *cdc9-1*, pBM272-*gal-cdc9-1* | This study  This study  This study  This study |
| ABy1605 | *cdc9-1* *pol30::pol30K164R (LEU2)*, pRS313 | This study |
| ABy1606 | *cdc9-1* *pol30::pol30K164R (LEU2)*, pRS313-*CDC9* | This study |
| ABy1607 | *cdc9-1 pol30::pol30K164R (LEU2)*, pRS313-*cdc9-K419A* | This study |
| ABy1608 | *cdc9-1 pol30::pol30K164R (LEU2)*, pRS313-*cdc9-K598A* | This study |
| ABy1609 | *cdc9-1 pol30::pol30K164R (LEU2)*, pRS313- *cdc9-NΔ60* | This study |
| ABy872  ABy1914  ABy1915  ABy1916 | *cdc9-1* pol30::pol30K107R (LEU2)*  *cdc9-1* pol30::pol30K107R (LEU2)*, pBM272-*gal*  *cdc9-1* pol30::pol30K107R (LEU2)*, pBM272-*gal-cdc9-1* Cl. 3  *cdc9-1* pol30::pol30K107R (LEU2)*, pBM272-*gal-cdc9-1* Cl. 7 | [3]  This study  This study  This study |
|  | **RDKY3615-derived strains** |  |
| RDKY3615 | *ura3-52, leu2∆1, trp1∆63, his3∆200, lys2∆Bgl, hom3-10, ade2∆1, ade8, htx13::URA3* | [5] |
| RDKY3735 | *sml1::KanMX, mec1::HIS3* | [6] |
| YKJM5789 | *pol30::HIS3*, YCPlac22-*POL30* | This study |
| YKJM5799 | *pol30::HIS3*, YCPlac22-*pol30-K183R* | This study |
| YKJM5985 | *pol30::HIS3*, YCPlac22-*pol30-K107R* | This study |
| YKJM5986 | *pol30::HIS3*, YCPlac22-*pol30-K117R* | This study |
| YKJM5988 | *pol30::HIS3*, YCPlac22-*pol30-K127R* | This study |
| YKJM5989 | *pol30::HIS3*, YCPlac22-*pol30-K127/164R* | This study |
| YKJM5991 | *pol30::HIS3*, YCPlac22-*pol30-K164R* | This study |

**REFERENCES**

1. Labib K, Tercero JA, Diffley JF (2000) Uninterrupted MCM2-7 function required for DNA replication fork progression. Science 288: 1643-1647.

2. Dornfeld KJ, Livingston DM (1991) Effects of controlled RAD52 expression on repair and recombination in Saccharomyces cerevisiae. Mol Cell Biol 11: 2013-2017.

3. Das-Bradoo S, Nguyen HD, Wood JL, Ricke RM, Haworth JC, et al. (2010) Defects in DNA ligase I trigger PCNA ubiquitylation at Lys 107. Nat Cell Biol 12: 74-79.

4. Ireland MJ, Reinke SS, Livingston DM (2000) The impact of lagging strand replication mutations on the stability of CAG repeat tracts in yeast. Genetics 155: 1657-1665.

5. Chen C, Kolodner RD (1999) Gross chromosomal rearrangements in Saccharomyces cerevisiae replication and recombination defective mutants. Nat Genet 23: 81-85.

6. Myung K, Datta A, Kolodner RD (2001) Suppression of spontaneous chromosomal rearrangements by S phase checkpoint functions in Saccharomyces cerevisiae. Cell 104: 397-408.
